# Insights from Experiment and Theory on Peculiarities of the Electronic Structure and Optical Properties of the Tl_2_HgGeSe_4_ Crystal

**DOI:** 10.1021/acs.inorgchem.3c01756

**Published:** 2023-10-04

**Authors:** Tuan V. Vu, Oleg Khyzhun, Galyna L. Myronchuk, Mariana Denysyuk, Lyudmyla Piskach, Andrij O. Selezen, Ilona Radkowska, Anatolii O. Fedorchuk, Svitlana S. Petrovska, Vira A. Tkach, Michał Piasecki

**Affiliations:** † Laboratory for Computational Physics, Institute for Computational Science and Artificial Intelligence, Van Lang University, 70000 Ho Chi Minh City, Vietnam; ‡ Faculty of Mechanical—Electrical and Computer Engineering, School of Technology, Van Lang University, 70000 Ho Chi Minh City, Vietnam; § Frantsevych Institute for Problems of Materials Science, National Academy of Sciences of Ukraine, 3 Krzhyzhanovsky Street, 03142 Kyiv, Ukraine; ∥ Department of Experimental Physics and Information-Measuring Technology, Lesya Ukrainka Volyn National University, 13 Voli Avenue, 43025 Lutsk, Ukraine; ⊥ Department of Chemistry and Technology, Lesya Ukrainka Volyn National University, 13 Voli Avenue, 43025 Lutsk, Ukraine; # Jan Dlugosz University in Częstochowa, Armii Krajowej 13/15, PL-42-217 Częstochowa, Poland; ¶ Department of Inorganic and Organic Chemistry, Lviv National University of Veterinary Medicine and Biotechnologies, 50 Pekarska Street, 79010 Lviv, Ukraine; ∇ Inorganic Chemistry Department, Uzhhorod National University, 46 Pidhirna, UA-88000 Uzhhorod, Ukraine

## Abstract

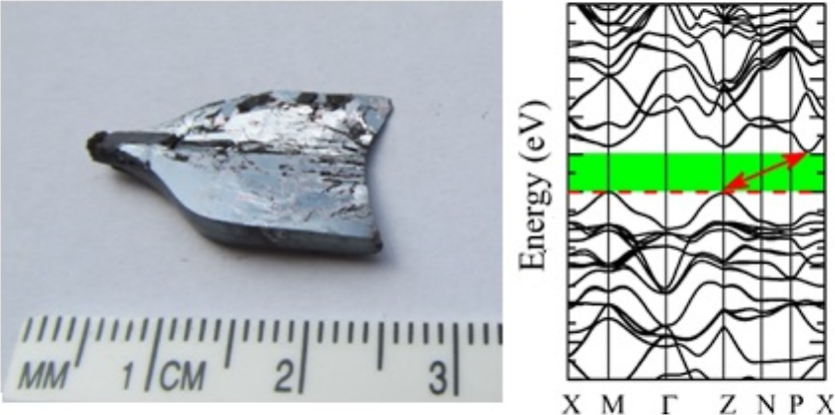

Tl_2_HgGeSe_4_ crystal was successfully, for the first time, synthesized by the Bridgman–Stockbarger technology, and its electronic structure and peculiarities of optical constants were investigated using both experimental and theoretical techniques. The present X-ray photoelectron spectroscopy measurements show that the Tl_2_HgGeSe_4_ crystal reveals small moisture sensitivity at ambient conditions and that the essential covalent constituent of the chemical bonding characterizes it. The latter suggestion was supported theoretically by ab initio calculations. The present experiments feature that the Tl_2_HgGeSe_4_ crystal is a high-resistance semiconductor with a specific electrical conductivity of σ ∼ 10^–8^ Ω^–1^ cm^–1^ (at 300 K). The crystal is characterized by p-type electroconductivity with an indirect energy band gap of 1.28 eV at room temperature. It was established that a good agreement with the experiments could be obtained when performing first-principles calculations using the modified Becke–Johnson functional as refined by Tran–Blaha with additional involvement in the calculating procedure of the Hubbard amendment parameter U and the impact of spin–orbit coupling (TB-mBJ + *U* + SO model). Under such a theoretical model, we have determined that the energy band gap of the Tl_2_HgGeSe_4_ crystal is equal to 1.114 eV, and this band gap is indirect in nature. The optical constants of Tl_2_HgGeSe_4_ are calculated based on the TB-mBJ + *U* + SO model.

## Abstract 2

Tl_2_HgGeSe_4_ crystal was successfully synthesized by the Bridgman–Stockbarger technology, and its electronic structure and optical properties were investigated. The crystal reveals small moisture sensitivity at ambient conditions and p-type electroconductivity with an indirect energy band gap of 1.28 eV at room temperature. A good agreement with the experiments could be obtained when performing first-principles calculations within the TB-mBJ + *U* + SO model.

## 1 Introduction

Quaternary copper-bearing sulfides and selenides of Cu_2_B^II^D^IV^X_4_ type (B^II^ represents zinc, cadmium, and mercury; D^IV^ is silicon, germanium, or tin; while X stands for sulfur and selenium) attract enormous attention during the recent two decades from engineering and scientific outlooks because they feature numerous prospective practical applications. Many sulfides and selenides of Cu_2_B^II^D^IV^X_4_ type demonstrate p-conductivity with band gap values being in the energy range of 1.0–1.56 eV, significant absorption coefficient values exceeding 10^4^ cm^–1^, rather big conversion power, etc.^1−11^ The above physicochemical properties make the Cu_2_B^II^D^IV^X_4_-type chalcogenides, in many cases, germanium-containing sulfides and selenides, very attractive compounds for practical use as effective absorbers for new-generation photovoltaic thin-film solar cell technologies,^12−15^ prospective thermoelectric materials,^16−18^ photocatalysts of conversion reactions,^19^ and semiconductors with promising electrical transport properties.^20,21^ Many Cu_2_B^II^D^IV^X_4_-type compounds crystallize in noncentrosymmetric structures; therefore, they attract attention as efficient nonlinear optical semiconductors.^22^ The important advantage of Cu_2_B^II^D^IV^X_4_-type chalcogenides is that a number of their physical and chemical properties, in particular, photovoltaic, transport, and thermoelectric behaviors, can be efficiently tuned to gain wishful technological magnitudes through doping them with other atoms,^20,23^ synthesis of solid solutions,^24−26^ formation of peculiar point vacancies and intrinsic defects,^15,27−29^ changing the dimensions of the crystals to nanosize values by formation of nanocrystals with controlled compositions,^30,31^ nanowire arrays,^13,32^ and nanorods.^19^ In particular, Cheng et al. have established recently in ref (33) that application of Cu_2_ZnGeS_4_ nanocrystals as a hole transport semiconductor for carbon-bearing perovskite-like solar cells allows to reach a maximum power conversion efficiency of 18.02% that is very beneficial for practical use. In addition, the physical and chemical properties of the quaternary Cu-bearing chalcogenides under discussion can be varied through phase transformations^34^ and inclusion of metastable and secondary phases.^35−37^ Furthermore, the annealing treatment is generally used for better controlling the secondary/metastable phase formation in the Cu_2_B^II^D^IV^X_4_ chalcogenides and improving the crystalline quality and thickness of thin-film absorbers based on them.^38−40^ This treatment also gives possibility for obtaining a particular layer morphology of such chalcogenides to gain demands of wide-gap film devices.^41^

In a recent decade, it was established that copper in the Cu_2_B^II^D^IV^X_4_-type compounds crystallizing generally in tetragonal (*I*4®2*m* and *I*4̅) or orthorhombic (*Pmn*2_1_ and *Cmc*2_1_) space groups^42^ can be substituted by thallium. This is due to the fact that copper belonging to group 11 of the periodic table possesses one s-electron on top of its filled d-shells, while Tl belonging to group 13 of the periodic table has three valence electrons (6s^2^ 6p^1^), but it reveals the inert Tl 6s^2^ electronic pair effect. Therefore, in some cases, thallium behaves like copper with respect to formation of quaternary chalcogenides.^43,44^ However, the Tl_2_B^II^D^IV^X_4_-type family is rather scarce. In particular, Mozolyuk et al. have established the formation of quaternary Tl_2_HgSn(Ge)Se_4_ selenides in the near-ternary systems Tl_2_Se–HgSe(Ge)–Sn(Ge)Se_2_,^45,46^ while Tl_2_HgSnS_4_ sulfide is formed in the system Tl_2_S–HgS–SnS_2_.^47^ Additionally, quaternary Tl_2_CdSn(Ge)Se_4_ selenides are found to form in the near-ternary system Tl_2_Se–CdSe(Ge)–Sn(Ge)Se_2_.^48^ All the above-mentioned thallium-containing quaternary sulfides and selenides Tl_2_B^II^D^IV^X_4_ (where B^II^ stands for Cd and Hg; while D^IV^ is Si, Ge, and Se; whereas X denotes S and Se) isostructurally crystallize in a noncentrosymmetric tetragonal space group *I*4®2*m*.^45−48^ Due to the results of the studies of phase equilibrium in the near-ternary system Tl_2_Se–HgGe–GeSe_2_,^46^ in which the quaternary selenide under consideration is formed, Tl_2_HgGeSe_4_ melts congruently at 764 K. After synthesis, the Tl_2_HgGeSe_4_ compound keeps its crystal structure and physicochemical properties under ambient conditions.

Following the fact that the detailed insight on the electronic band structure is a primary key for predicting suitable routes of modification of the physical/chemical properties of solids to a desired technological request, the electronic band structure computing data and/or X-ray spectroscopy investigations were gained recently for Tl_2_HgSnS_4_ and Tl_2_B^II^D^IV^Se_4_ (B^II^ = Cd and Hg and D^IV^ = Ge and Sn) compounds.^49−52^ These results allow for a statement that the contribution of electronic p-like states associated with S(Se) atoms prevails in their valence band area, and contributions of Tl 6s states prevail near the valence band bottom. The Tl_2_HgSnS_4_ and Tl_2_B^II^D^IV^Se_4_ (B^II^ = Cd and Hg; D^IV^ = Ge and Sn) chalcogenides are direct gap materials.^49−52^

Because of the relative novelty of Tl_2_HgGeSe_4_ selenide, as the literature data feature, peculiarities of its electronic band structure and optical constants, to the best of our knowledge, have not yet been investigated, both theoretically and experimentally. To overcome this lack, we have made experimental as well as theoretical studies of the electronic and optical properties of the titled selenide. Gaining our tasks to clarify the peculiarities of the electronic band structure of Tl_2_HgGeSe_4_, we apply the most accurate techniques as carried out in the WIEN2k program.^53^ Aiming to verify the data of the present theoretical findings, we use measurements of a Tl_2_HgGeSe_4_ crystal which, for the first time, was synthesized in the present work by the Bridgman–Stockbarger growth technique to explore the peculiarities of filling its valence band area by electronic states related to constituent atoms. Such measurements were made using the X-ray photoelectron spectroscopy (XPS) technique. The XPS method was also applied to investigate the charge states of the constituting atoms and to compare the peculiarities of the chemical bonding of Tl_2_HgGeSe_4_ selenide with those of its quaternary Ge-containing counterparts. The energy distributions of some of the most important contributors in the valence band area of the Tl_2_HgGeSe_4_ crystal were studied using the possibility of X-ray emission spectroscopy (XES). We involve different models aiming to achieve the finest correspondence between theory and experiments. When achieving the finest theoretical technique available to reproduce most accurately the present experimental findings, we calculate in detail the main optical properties of the Tl_2_HgGeSe_4_ crystal. To explore the vibration modes of the Tl_2_HgGeSe_4_ crystal, we use measurements of the Raman spectra employing two different laser excitations, at 532 and 830 nm.

## 2 Experimental Section

**Figure 1:**
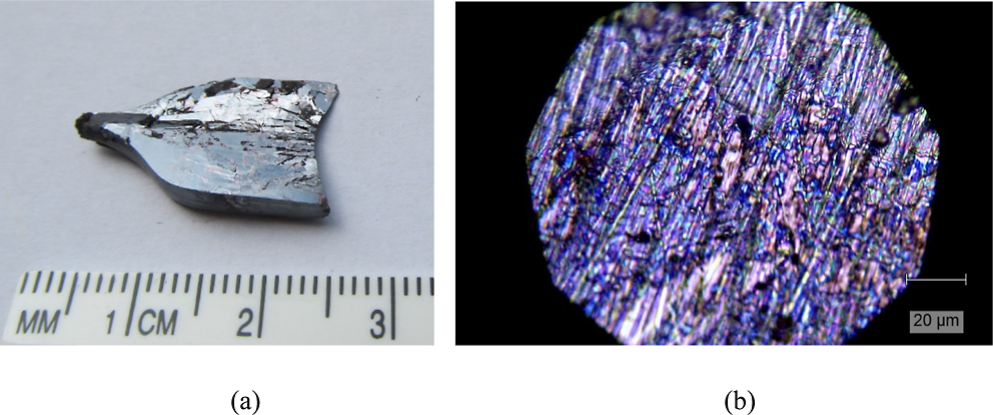
(a) Photo of a piece cleaved from the Tl_2_HgGeSe_4_ crystal grown using the Bridgman–Stockbarger method and (b) its surface morphology.

In the present experiments on X-ray spectroscopy and measurements of the optical coefficient of absorption and Raman spectra, we deal with a Tl_2_HgGeSe_4_ crystal grown by the Bridgman–Stockbarger method. Figure 1a demonstrates a photo of a piece of the as-grown Tl_2_HgGeSe_4_ crystal used in the present measurements. Its centimeter-size dimensions allow for practical applications. The crystal growth conditions were chosen from the analysis of the *T*–*x* diagram of the Tl_2_GeSe_3_–HgSe system.^46^ The process of growth of single crystals was carried out using a special furnace consisting of two temperature zones with independent temperature regulation. The temperature gradient in the crystallization area was set to be within 4–6 K/mm, the temperature of the upper “hot” zone of the growth furnace was chosen to be 900 ± 20 K, the lower “cold” zone was 500 ± 20 K, while the growth rate was 0.2 mm/h. After crystallization of the melt, annealing was carried out for over 100 h. Cooling to room temperature was made at a rate of 20–30 K/h. In accordance with the data of X-ray diffraction analysis, the crystal under consideration is a single-phase Tl_2_HgGeSe_4_ compound with unit cell constants *a* = 7.9984(2) Å and *c* = 6.7645(2) Å and the structure belonging to the tetragonal space group *I*4̅2*m* (Table S1).

These crystal parameters are in fair agreement with those detected in ref (46) for the Tl_2_HgGeSe_4_ alloy (Table 1). From the studies of surface morphology (Figure 1b), one can state that the crystal consists of many single crystals differing in “color” changing from light pink, through green, to dark blue, and violet. The single crystals have a preferable direction of growth, and they are usually needle shaped; the pink crystals which are random shaped, mainly block shaped, and have the largest area among all the types of single crystals are the only exception. The dominating “colors” of the single crystals are navy blue and pink. Quite interesting is the right upper side of the studied surface: there are some crystals formed with layers of different “colors”, which resembles iridescence. The surface of the crystal sample is not flat, which is associated with the presence of a different number of single-crystal layers and their size. The surface is not smooth: there are some holes and black objects randomly localized. The crystal most likely has a layered structure. In addition, the detected play of color on the surface (iridescence and interference) can be due to the manifestation of layers of different thicknesses.

**Table 1:** Optimized Unit Cell Parameters and the Atomic Positions of Tl_2_HgGeSe_4_ (Space Group *I*4®2*m*) in Relation to the Experiments

cell parameters	experiments	the present computing results
*a* (Å)	7.9947(4)^a^/7.9984(2)^b^	8.07448
*c* (Å)	6.7617(4)^a^/6.7645(2)^b^	6.91823

atomic coordinates							
	Wyckoff site	*x/a*	*y/b*	*z/c*	*x/a* opt	*y/b* opt	*z/c* opt
Tl	4c	0.0^a^^,^^b^	0.5^a^^,^^b^	0.0^a^^,^^b^	0.0	0.5	0.0
Hg	2b	0.0^a^^,^^b^	0.0^a^^,^^b^	0.5^a^^,^^b^	0.0	0.0	0.5
Ge	2a	0.0^a^^,^^b^	0.0^a^^,^^b^	0.0^a^^,^^b^	0.0	0.0	0.0
Se	8i	0.1693(2)^a^/0.16558(8)^b^	0.1693(2)^a^/0.16558(8)^b^	0.2189(5)^a^/0.2164(2)^b^	0.16922	0.16922	0.21636

a Ref (46), Tl_2_HgGeSe_4_ alloy.

b Present work, Tl_2_HgGeSe_4_ crystal.

In the present X-ray spectroscopy measurements of the Tl_2_HgGeSe_4_ crystal, we generally follow techniques that we used previously for the analogous studies of the electronic structure of its cadmium-bearing counterpart, Tl_2_CdGeSe_4_.^52^ In brief, we have derived the XPS spectra by employing the UHV analysis system (SPECS, Berlin, Germany). The spectra were excited by using a Mg Kα X-ray source (*h*ν = 1.2536 keV) and measured at a constant pass energy mode, which was equal to 28 eV. The system energy scale was calibrated by employing reference pure etalon gold and copper metals as reported elsewhere.^54^ It should be noted that, in the case of XPS studies of Cu(Tl)_2_B^II^D^IV^X_4_-type sulfides and selenides, the charging surface effects are generally overcome by measuring the reference core-level C 1s line originated from a thin layer of adsorbed hydrocarbon by adjusting its binding energy (BE) to 284.6 eV.^49,50^ However, when employing a Mg Kα X-ray source to excite the XPS spectra of Ge-bearing compounds, there is a superposition of the hydrocarbon C 1s spectrum with the neighboring auger Ge L_2_M_23_M_23_ line.^55^ As a result, in the present experiments, the charging surface effect has been compensated by using a specially constructed flood gun available in the SPECS UHV analysis system as we used to do such a technique in the case of Ge-bearing Cu(Tl)_2_B^II^D^IV^X_4_-type chalcogenides.^5,34,52^ Following the findings on the electronic structure of quaternary Ge-bearing selenides Cu(Tl)_2_B^II^GeSe_4_ indicating that among electronic states associated with these atoms one could expect essential input of Se(Ge) 4p-like states in the valence band area,^4,5,52^ we have recorded the fluorescent XES Se(Ge) Kβ_2_ spectra originating due to the transition from the N_II,III_ shell to the K level and supplying information regarding the peculiarities of energy distributions of valence Se(Ge) p-like electronic states.^56,57^ The latter bands were derived with a DRS-2 M spectrometer in the third order of reflection and employing for spectral excitation a BHV-7 X-ray tube with a Au anode. The operating conditions of the DRS-2 M spectrometer are completely the same as they were chosen for the similar XES experiments of related Cd-containing selenide, Tl_2_CdGeSe_4_.^52^ The edge of the optical absorption of the Tl_2_HgSnSe_4_ crystal was measured using an MDR-206 monochromator with the spectral resolution not worth than 0.2 nm using the technique reported in ref (49). The peculiarities of spectral allocation of photoconductivity in the Tl_2_HgGeSe_4_ crystal were estimated following the technique^49^ by measuring the electrical response by employing a Keithley 6514 electrometer (the noise level is lesser than 1 fA). As a material for electrical contacts, we used gallium–indium eutectic. The contact ohmic resistance was verified prior to the experiment, and the accuracy of recording resistance was estimated to be within 1.5%.

Raman spectroscopy measurements were performed employing a Renishaw In Via Spectrometer 3RTG68 equipped with a Renishaw Centrus 3CMC21 detector (objective: ×50) and using two laser excitation wavelengths: 532 and 830 nm. In particular, the operation conditions of the laser emitting at 532 nm and covering the spectral range from 48.65 to 4000.05 cm^–1^ are as follows: power: 0.25%, grating: 2400 lines/mm, exposure time: 60.0 s, and five subsequent accumulations. However, the operation conditions of the laser emitting at 830 nm and covering the spectral range from 94.6 to 4000 cm^–1^ edge are as follows: line focus mode, power: 0.05%, grating: 1200 lines/mm, exposure time: 1.0 s, and 100 subsequent accumulations. All the measurements were carried out at room temperature. The spectral parameters were adapted experimentally to obtain the finest quality of the Raman spectra with a high value of the peak-to-noise level ratio. For the map surface measurements, we used the excitation of the 830 nm laser (32 acquisitions (16 × 2 points), 1 s exposure time, two subsequent accumulations, 0.1% laser power, and spectral range: 94.6–4000 cm^–1^ Raman shift).

## 3 Crystal Structure and First-Principles Calculations

**Figure 2:**
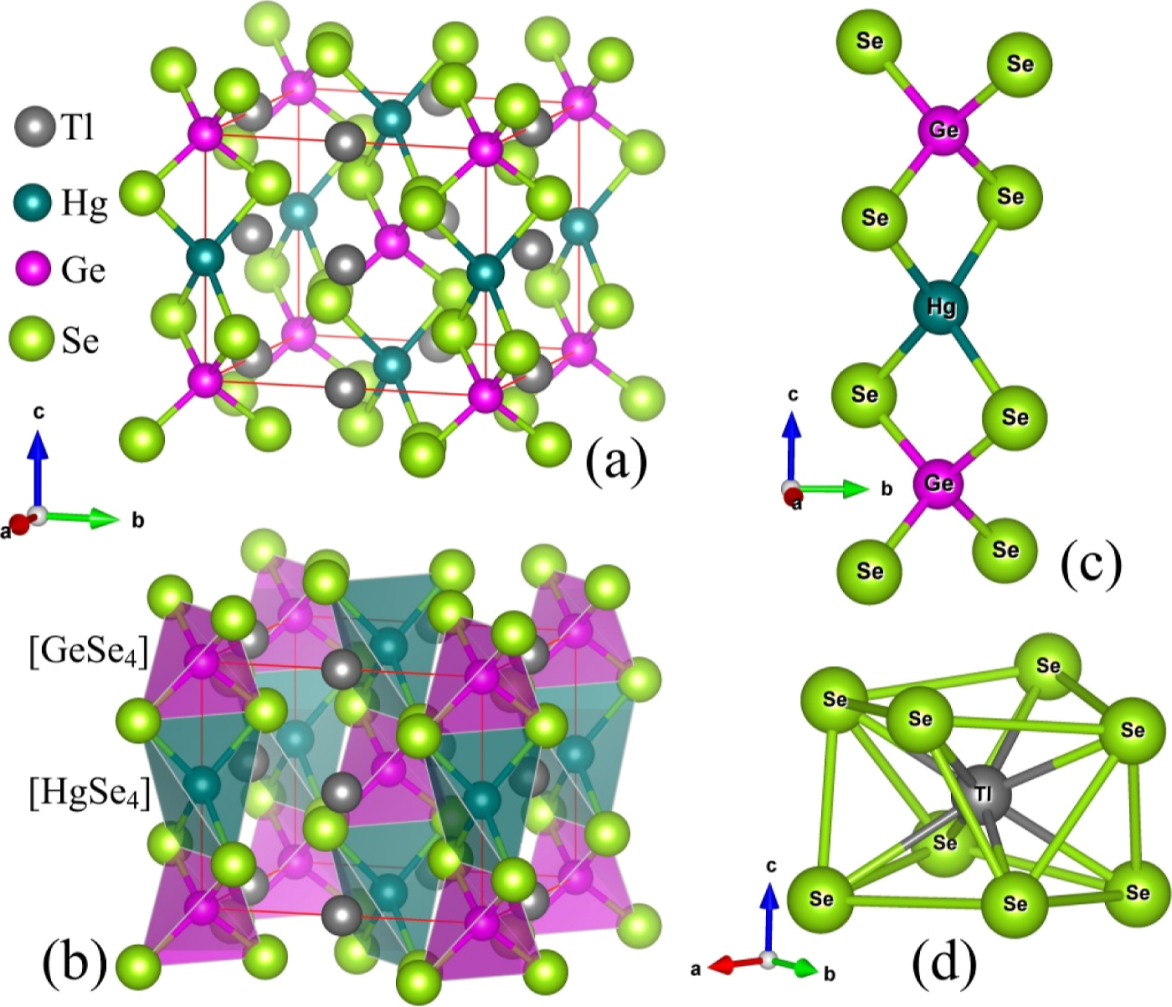
(a) Atomic arrangement and (b) stacking of [GeSe_4_] and [HgSe_4_] tetrahedra in the Tl_2_HgGeSe_4_ structure (note: the unit cell is outdrawn), the nearest surrounding of (c) germanium and mercury atoms, and (d) thallium atoms in the quaternary selenide under consideration.

The present first-principles calculations are made within the density functional theory (DFT), and they were carried out using the augmented plane wave plus local orbital method as realized in WIEN2k software.^53^ In the computations, for the atoms composing the Tl_2_HgSnSe_4_ crystal, we use muffin-tin (MT) spherical radii as follows: thallium—2.50 au, mercury—2.39 au, germanium—2.05 au, and selenium—2.05 au. In the present computing procedure, we employ the initial lattice constants *a* = 7.9947 Å and *c* = 6.7617 Å and atom positions in the unit cell (see the data of Table 1) as they have been established experimentally for Tl_2_HgGeSe_4_ by Mozolyuk et al.^46^ Then, after the procedure of structure optimization, we gain the unit cell constants *a* = 8.07448 Å and *c* = 6.91823 Å and atom positions being in good correspondence with the experimental findings obtained in ref (46) and in the present work (Table 1). We have used semicore + valence electron configurations in the present calculation procedure as follows: thallium (5p^6^5d^10^6s^2^6p^1^), mercury (5p^6^5d^10^6s^2^), germanium (3d^10^4s^2^4p^2^), and selenium (3d^10^4s^2^4p^4^). The core electrons of the atoms constituting the Tl_2_HgGeSe_4_ compound were treated in the DFT calculations, as well. Particularly, the present results feature that the differences between the experimentally^46^ established and theoretically optimized lattice constants *a* and *c* are less than 1.0 and 2.3%, respectively. Furthermore, the theoretical and experimental lengths of the Tl(Hg,Ge)–Se bonds of Tl_2_HgGeSe_4_ are in fair agreement with each other as the data collected in Table 2 reveal. It is necessary to note that the Tl_2_HgGeSe_4_ crystal structure (Figure 2a) belonging to tetragonal SG (*I*4̅2*m*) is noncentrosymmetric. This fact may suggest good prospective for the use of this quaternary selenide in nonlinear optics. In this structure, Tl, Hg, Ge, and Se atoms are positioned at 4c, 2b, 2a, and 8i Wyckoff positions, respectively. There exist four Se atoms in the nearby surroundings of germanium and mercury atoms forming tetrahedra [GeSe_4_] and [HgSe_4_], respectively (see Figure 2d). The tetrahedra [GeSe_4_] and [HgSe_4_] are stacked in the Tl_2_HgGeSe_4_ unit cell as shown in Figure 2b. With respect to thallium atoms, in the Tl_2_HgGeSe_4_ structure, they possess tetragonal antiprismatic surroundings created by selenium atoms (Figure 2d).

**Table 2:** Calculated Bond Lengths in Comparison with the Experimentally Established Ones^46^

bonds	calculated in this work (Å)	measured experimentally^46^ (Å)
Tl–Se	3.3528	3.323(3)
	3.5848	3.523(2)
Hg–Se	2.7540	2.688(2)
Ge–Se	2.4443	2.420(2)

With respect to peculiarities of the present first-principles calculating procedure, we use different models for such a case because commonly applied local density approximation in the form of Ceperley–Alder^58^ or generalized gradient approximation (GGA) in the presentation developed by Perdew–Burke–Ernzerhof (GGA-PBE)^59^ treated for exchange–correlation (XC) potential generally cause big lack of the energy band gaps, *E*_g_, and insufficient correspondence of the energy allocation of electronic states in the vicinities of the valence band area, mostly just below the main part of the valence band, of semiconducting materials, in particular quaternary (Cu,Tl)_2_B^II^D^IV^X_4_-type sulfides and selenides.^6,34,50,52,60^ As the above achievements in ab initio band structure computations of such compounds indicate, the use of modified Becke–Johnson (mBJ) functional in the presentation developed by Tran–Blaha^60^ and containing the Hubbard amendment parameter *U* and spin–orbit (SO) coupling (TB-mBJ + *U* + SO model) gives the best agreement with the experiments in the *E*_g_ value and peculiarities of the occupation of the electronic states within the broad valence band area. Therefore, we verify here the application of different models aiming to achieve the best agreement of the theoretical and experimental findings for Tl_2_HgGeSe_4_ selenide.

It is worth mentioning that the most important computing parameters *R*_min_^MT^*k*_max_ = 8 (*R*_min_^MT^ and *k*_max_ are the smallest MT spherical radius and the biggest dimension of the *k* vector value in the plane wave expansion, respectively) and *G*_max_ = 14 (a.u.)^−1^ (magnitude of charge density Fourier expansion) are employed in the given DFT calculations. We used a grid amounting to 1000 *k*-points for Brillouin zone sampling via its irreducible wedge. The computing iterations were carried out until reaching the condition *q* = ∫|ρ_*n*_ – ρ_*n*–1_| d*r* ≤ 10^–4^, where ρ_*n*_(*r*) and ρ_*n*–1_(*r*) are ascribed to the charge density of the present and previous iterations, respectively, as it is generally proposed to follow in the case of (Cu,Tl)_2_B^II^D^IV^X_4_-type sulfides and selenides.^6,34,50,52,61^

When reaching the best coincidence of the given theoretical data with the experimental measurements regarding features of the energy allocation of electronic states in the vicinities of the valence band area of Tl_2_HgGeSe_4_ and *E*_g_ value, then, we calculate the optical constants of this compound using a 5000 *k*-point grid. In particular, the imaginary portion of the complex dielectric function is expressed by the following equation
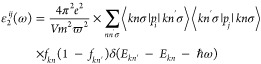
1where *e* and *m* are, respectively, the charge of electron and its mass, ω presents the electromagnetic (EM) wave angular frequency, *V* denotes a volume of the unit cell, *p* is the momentum operator, |*kn*σ⟩ is the crystal wave function, *k* denotes the wave vector, and σ stands for the spin associated with the energy eigenvalue, *E*_*kn*_.

The real component ε_1_ (ω) of the complex dielectric function could be derived using the expression for the imaginary component ε_2_ (ω) of the dielectric function following Kramers–Kronig’s relation
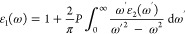
2where *P* presents the main value of the integral.

Such optical constants as α(ω) (optical absorption coefficient), *n*(ω) (refractive index), *k*(ω) (extinction coefficient), *R*(ω) (optical coefficient of reflectivity), and *L*(ω) (spectrum of electron energy loss) could be obtained from real and imaginary portions, ε_1_ (ω) and ε_2_ (ω), of the dielectric function via equations^62^
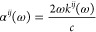
3
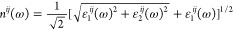
4
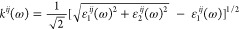
5
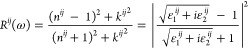
6
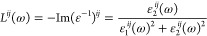
7

The birefringence Δ*n*(ω) is expressed as the difference between the extraordinary and ordinary indexes of refraction. It is worth mentioning that the DFT method was proven to be among the most accurate techniques for the computations of the electronic structure of solids.^63−69^

## 4 Results and Discussion

### 4.1 XPS Tests of the Tl_2_HgGeSe_4_ Crystal

**Figure 3:**
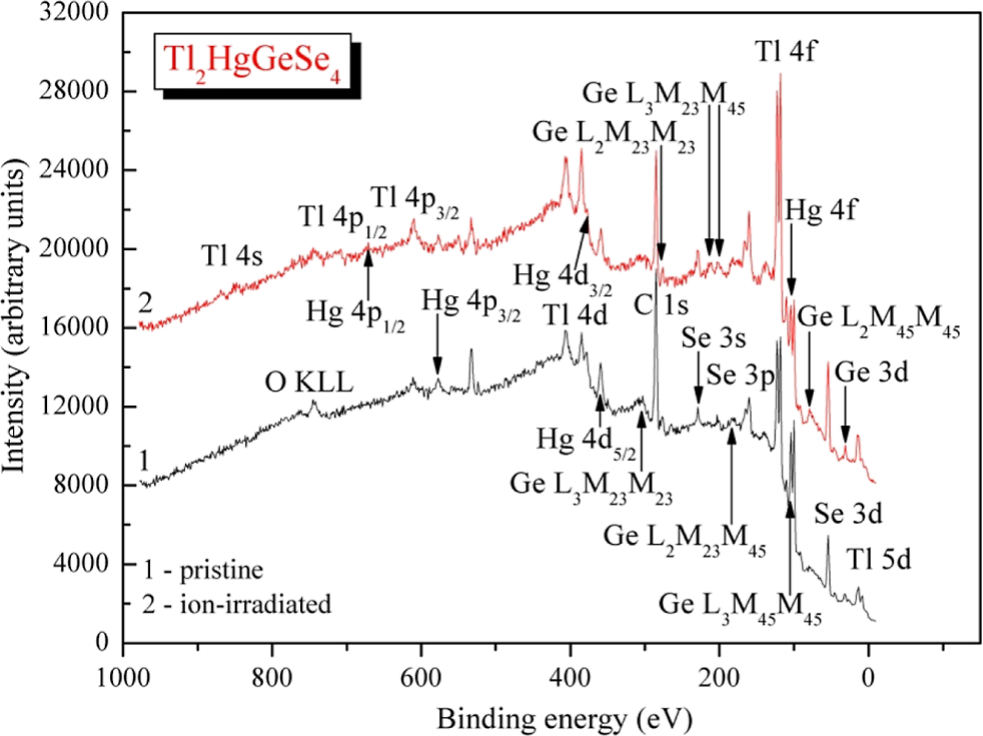
XPS survey spectra of Tl_2_HgGeSe_4_: (1) as-synthesized crystal surface and (2) surface after its 3 kV Ar^+^ ion-beam-induced treatment.

The results of XPS studies of the Tl_2_HgGeSe_4_ crystal are shown in Figure 3. The XPS measurements performed in a wide-scale region (Figure 3) show that the spectral features of the as-synthesized Tl_2_HgGeSe_4_ crystal surface are those belonging to the constituting atoms except for the core-level spectra caused by hydrocarbon peculiarities or oxygen-including species that have been adsorbed on the as-synthesized crystal surface due to its contact with laboratory air prior the present experiments were carried out (in fact, a couple of days). We do not see any formations of carbonate- or oxide-forming species because the C 1s and O 1s lines (not demonstrated here) are viewed as small-intensive diffusive spectra with their maxima positioned at binding energies near 284.58 and 532.06 eV, respectively. These species are very weakly bonded with the crystal surface because the 3 kV Ar^+^ ion-beam-induced treatment over 5 min brings to abrupt decreasing of the relative intensities of the C 1s and O 1s core-level spectra caused by hydrocarbon peculiarities or oxygen-including species (Figure 3, curve 2). The use of 3 kV Ar^+^ ion-beam-induced treatment over 5 min is motivated by the fact that such operating conditions of surface cleaning were found to be optimal for similar studies of the chemical stability of related Ge-bearing quaternary Cu_2_B^II^GeS(Se)_4_ chalcogenides (B^II^ = Zn, Cd, and Hg).^4,5,70,71^ The use of the same operating conditions is very important for correct comparing the effect of influence of Ar^+^ ion-beam-induced treatment on the stability of these compounds. Our previous XPS experiments indicate that a more prolonged treatment of the Cu_2_B^II^GeS(Se)_4_ chalcogenides with 3 kV Ar^+^ ions is accompanied by possible partial surface amorphization and embedding Ar atoms in the top surface analyzing layers. Therefore, the present XPS data reveal that the Tl_2_HgGeSe_4_ crystal surface possesses rather small moisture sensitivity, which is also a specific peculiarity of other quaternary Ge-containing sulfides and selenides, in particular Tl_2_CdGeSe_4_,^52^ Cu_2_HgGeS_4_,^70^ and Cu_2_HgGeSe_4_.^71^ This property of the Tl_2_HgGeSe_4_ surface might be rather useful for practical use of such crystals in devices that work under conditions of ambient atmosphere.

**Figure 4:**
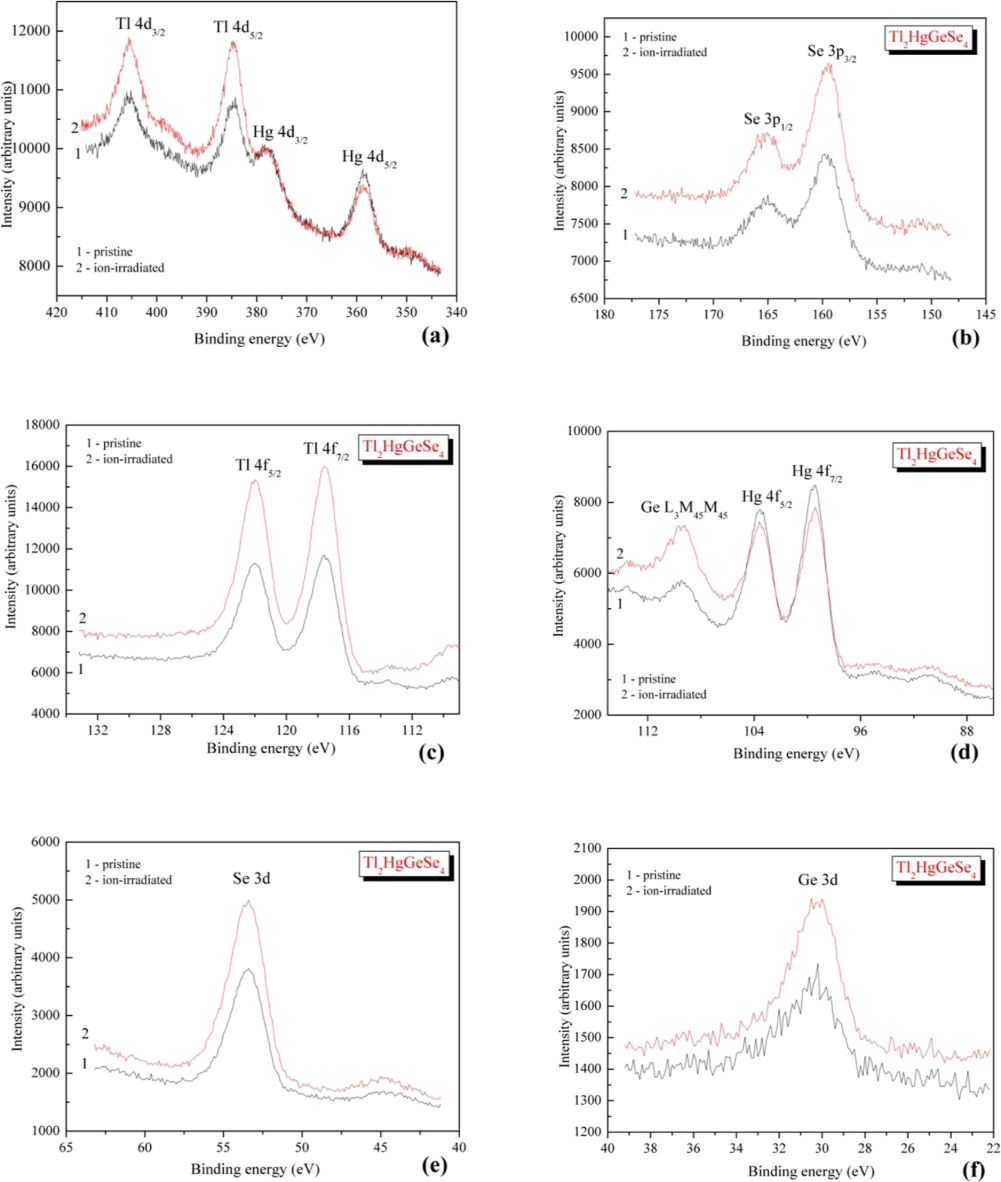
XPS core-level spectra of Tl_2_HgGeSe_4_ as recorded for (1) as-synthesized crystal surface and (2) surface after its 3 kV Ar^+^ ion-beam-induced treatment: (a) Tl(Hg) 4d, (b) Se 3p, (c) Tl 4f, (d) Hg 4f and Ge L_3_M_45_M_45_, (e) Se 3d, and (f) Ge 3d.

The detailed most informative core-level XPS spectra for composing atoms of the Tl_2_HgGeSe_4_ crystal are shown in Figure 4, while Table 3 presents data on measurements of BE values as recorded for the pristine surface and after its 3 kV Ar^+^ ion-beam-induced treatment. Following the total charge neutrality of the Tl_2_HgGeSe_4_ crystal, its atomic composition can be written as (Tl^+^)_2_Hg^2+^Ge^4+^(Se^2–^)_4_. Nevertheless, comparing the BE magnitudes as measured for core-level electrons related to the constituting atoms of the Tl_2_HgGeSe_4_ crystal with the literature data reported elsewhere,^55,72^ one can state that the charge states of thallium, mercury, and germanium atoms in the given crystal are smaller than expected to be +1, +2, and +4, respectively. These experimental data could be explained by assuming the fact that the covalent part (in addition to the ionic part) of the chemical bonding should be rather essential in the Tl_2_HgGeSe_4_ crystal.

**Table 3:** Binding Energies Measured in eV^a^ for Constituent Element Core-Level Electrons of As-Grown and Ar^+^ Ion-Irradiated Surfaces of the Tl_2_HgGeSe_4_ Single Crystal and of As-Grown Surfaces of Related Germanium-Bearing Quaternary Selenides

core-level or auger line	Tl_2_HgGeSe_4_/as-grownsurface	Tl_2_HgGeSe_4_/surface treated by Ar^+^ ions	Tl_2_CdGeSe_4_/as-grown surface	Cu_2_HgGeSe_4_/as-grown surface	Tl_2_HgSnSe_4_/as-grown surface
Tl 5d_5/2_	12.36	12.33	12.32		12.79
Tl 5d_3/2_	14.45	14.43	14.36		14.85
Ge 3d	30.36	30.28	30.19	30.14	
Se 3d	53.43	53.45	53.29	53.70	53.73
Hg 4f_7/2_	99.48	99.43		99.55	99.87
Hg 4f_5/2_	103.54	103.50		103.51	103.88
Ge L_3_M_45_M_45_	109.44	109.49		109.20	
Tl 4f_7/2_	117.57	117.53	117.56		118.02
Tl 4f_5/2_	122.01	121.96	122.07		122.43
Se 3p_3/2_	159.68	159.62		159.85	159.77
Se 3p_1/2_	165.17	165.14		165.41	165.49
Hg 4d_5/2_	358.62	358.69		358.8^b^	359.23
Hg 4d_3/2_	377.99	378.11		378.0^b^	
Tl 4d_5/2_	384.75	384.68			385.21
Tl 4d_3/2_	405.49	405.42			405.88
reference	present work	present work	(52)	(71)	(73)

a Precision of measurements is ±0.05 eV.

b Precision of measurements is ±0.1 eV.

**Figure 5:**
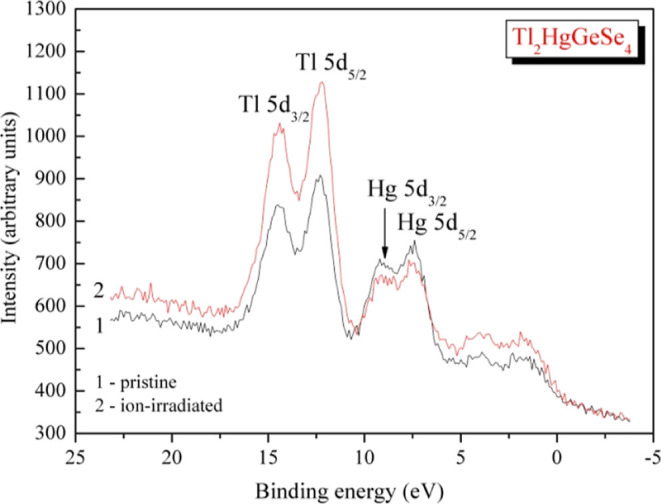
XPS spectra measured for the valence band area of Tl_2_HgGeSe_4_: (1) as-synthesized crystal surface and (2) surface after its 3 kV Ar^+^ ion-beam-induced treatment.

As could be noted from the XPS measurements plotted in Figure 4 and data listed in Table 3, the 3 kV Ar^+^ ion-beam-induced treatment does not cause changes in the BE magnitudes; however, this surface treatment accompanies enhancing the relative intensities of the XPS spectra related to all the constituting atoms except of mercury. This fact could be explained by etching Hg atoms in the top near-surface analyzing layers of the Tl_2_HgGeSe_4_ crystal caused by the mentioned treatment by 3 kV Ar^+^ ions. In fact, using the literature chemical element sensitivity factors^55^ and based on measurements of intensities of the XPS core-level thallium 4f_7/2_, mercury 4f_7/2_, germanium 3d, and selenium 3d electrons, one can state that the atomic composition (in at %) of the as-synthesized Tl_2_HgGeSe_4_ crystal is Tl/Hg/Ge/Se = 24.5/13.1/12.2/50.2 being in close relation to perfect stoichiometry Tl/Hg/Ge/Se = 25.0/12.5/12.5/50.0. After the 3 kV Ar^+^ ion-beam-induced treatment, the atomic composition is as follows: Tl/Hg/Ge/Se = 26.8/8.6/13.5/51.1. However, this substoichiometry does not evoke essential changes with respect to the binding energies of the core-level electrons of the constituting atoms as the measuring results listed in Table 3 present. The above fact indicates that 3 kV Ar^+^ ion-beam-induced treatment evokes decreasing of the mercury content within the top near-surface analyzing layers of the Tl_2_HgGeSe_4_ crystal by nearly 34.4%. Recent studies of the tin-bearing counterpart, Tl_2_HgSnSe_4_, have revealed^73^ that at the same operation conditions regarding the Ar^+^ ion-beam-induced treatment, we detected decreasing contents of Hg and Sn atoms in the topmost analyzing layers by about 17.3 and 28.0%, respectively. This means that with respect to decreasing the content of mercury atoms within the top near-surface analyzing layers due to bombardment by 3 kV Ar^+^ ions, we detect a similar behavior of the Tl_2_HgC^IV^Se_4_ (C^IV^ = Ge and Sn) crystals. However, the impact of this surface treatment with respect to the relative content of the C^IV^ element in the Tl_2_HgC^IV^Se_4_ crystals is quite different. The decrease of the content of mercury atoms within the top near-surface analyzing layers of the Tl_2_HgGeSe_4_ crystal is accompanied by changes in the shape of the valence electron energy allocations, mainly in the region relating to the area just below the main portion of the valence band (Figure 5). It is worth indicating that the effect of decreasing mercury content was found to be very pronounced in Cs_2_HgQ_4_ (Q = Cl, Br, and I) halides: the 3 kV Ar^+^ ion-beam-induced treatment, in some cases, leads to a decrease in its content in several times.^62,74,75^

It is well known that for evaluating the degree of ionicity of the chemical bonding, it is very helpful to deal with the XPS difference parameters Δ: the bigger parameter Δ value determined as the BE difference of the core-level electrons related to a cation and an anion, the higher onicity degree of the cation–anion bonds.^76^ Following the aim to evaluate comparatively the peculiarities of the chemical bonding of the Tl_2_HgGeSe_4_ crystal with those of related germanium-containing counterparts, we collected in Table 4 the results of determining the difference Δ_Tl_, Δ_Hg_, and Δ_Ge_ parameters that were calculated based on the differences of experimentally measured binding energies of Tl 4f_7/2_, Hg 4f_7/2_, and Ge 3d electrons, respectively, and Se 3d electrons. As one can notice from the data gathered in Table 4, the ionicity degrees of the chemical Ge–Se bonds are comparative in Tl_2_HgGeSe_4_ and Tl_2_CdGeSe_4_ selenides, and they are bigger in comparison with that in Cu_2_HgGeSe_4_. Further, the chemical Hg–Se bond ionicity degree enhances when going from Cu_2_HgGeSe_4_ to Tl_2_HgGeSe_4_ and, then, to Tl_2_HgSnSe_4_ because we detect increasing value of the Δ_Hg_ parameter from 45.85 ± 0.05 eV to 46.05 ± 0.05 eV and, then, to 46.14 ± 0.05 eV in the above sequence of quaternary selenides. Furthermore, we detect the comparative ionicity degrees of the chemical Tl–Se bonds in Tl_2_CdGeSe_4_ and Tl_2_HgSnSe_4_ compounds, and this degree decreases when going to Tl_2_HgGeSe_4_ (Table 4).

**Table 4:** Difference Parameters Determined with an Accuracy of ±0.05 eV in the Tl_2_HgGeSe_4_ Crystal and Related Germanium-Bearing Quaternary Selenides

difference parameter	Tl_2_HgGeSe_4_	Tl_2_CdGeSe_4_	Cu_2_HgGeSe_4_	Tl_2_HgSnSe_4_
Δ_Ge_^a^	–23.07	–23.10	–23.56	
Δ_Hg_^b^	46.05		45.85	46.14
Δ_Tl_^c^	64.14	64.27		64.29
Reference	present work	(52)	(71)	(73)

a Difference of the binding energies of the XPS Ge 3d and Se 3d core-level spectra.

b Difference of the binding energies of the XPS Hg 4f_7/2_ and Se 3d core-level spectra.

c Difference of the binding energies of the XPS Tl 4f_7/2_ and Se 3d core-level spectra.

### 4.2 Electronic Structure of the Tl_2_HgGeSe_4_ Crystal as Evidenced from the DFT Computation and XPS and XES Experiments

**Figure 6:**
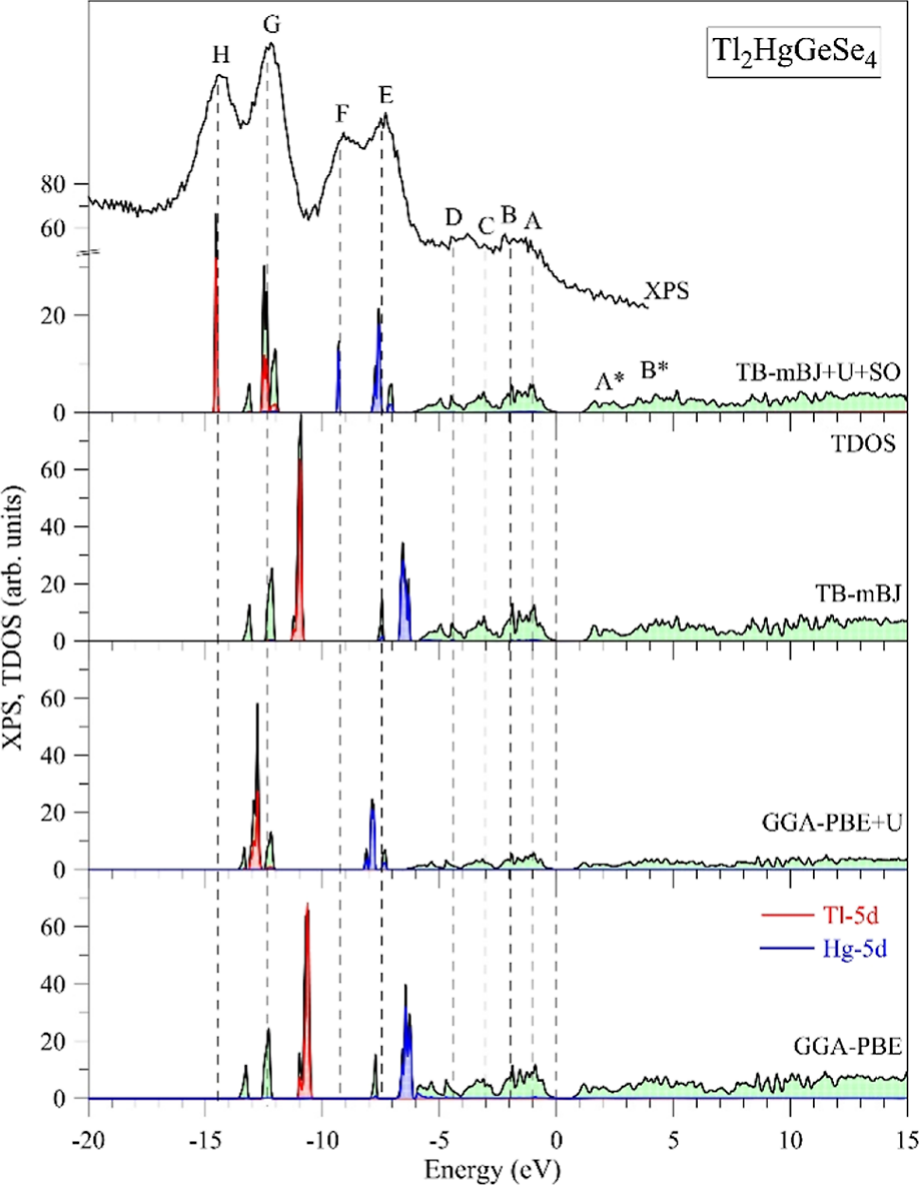
TDOS of Tl_2_HgGeSe_4_ calculating within different models (GGA-PBE, GGA-PBE + *U*, TB-mBJ, and TB-mBJ + *U* + SO) adjusted in a common energy scale with the XPS spectrum recorded for valence band area of the Tl_2_HgGeSe_4_ crystal (initial surface).

With the aim of evaluating peculiar features of population by electronic states of the valence band area of the Tl_2_HgGeSe_4_ crystal and the nature of its energy band gap, we carry out first-principles DFT computation of the electronic structure of this compound. Recent data dealing with DFT computation of several quaternary chalcogenides belonging to the Cu(Tl)_2_B^II^D^IV^X_4_-type series present^9,50,52,70,71^ that it is necessary to treat different models to achieve a good agreement of theoretical and experimental results. This fact is explained by the fact that the generally employed GGA-PBE technique fails in many cases when using for XC potential in calculations of the Cu(Tl)_2_B^II^D^IV^X_4_-type compounds. This is characteristic, in particular, in the case of the DFT computing results regarding Tl_2_HgGeSe_4_. Figure 6 displays the data of our computation of curves of total DOS (TDOS) using different approaches (in our case, the GGA-PBE, TB-mBJ, GGA-PBE + *U*, and TB-mBJ + *U* + SO models).

As could be noted from Figure 6, in the vicinity of the main area of the valence band (the energy area corresponding to peculiarities A–D), the features of the energy allocation of valence electronic states are roughly similar for all the models being used. However, essential differences can be seen for the electronic states located just at the bottom of the valence band (the energy area corresponding to peculiarities E–H). Figure 6 demonstrates that the use of the GGA-PBE model as well as the TB-mBJ model is accompanied by underestimations of the energy locations of Hg 5d electronic states by 1.1 and 1.0 eV, respectively, as compared to the experimental position of peculiarity E of the XPS spectrum. The underestimating values for the energy locations of Tl 5d electronic states (peculiarity G of the XPS spectrum) are about 1.4 and 1.2 eV when using in the DFT computation of the GGA-PBE model and the TB-mBJ model, respectively. As can be seen from Figure 6, in order to gain fair agreement of theory and experiment, we have to use other techniques in the DFT calculations. In particular, Figure 6 presents that the involvement in the GGA-PBE computation of the Hubbard amendment parameter *U* (GGA-PBE + *U* model) results in overestimations of the energy positions of Hg 5d and Tl 5d bands by nearly 0.5 and 0.8 eV, respectively, in comparison with the experiment.

**Figure 7:**
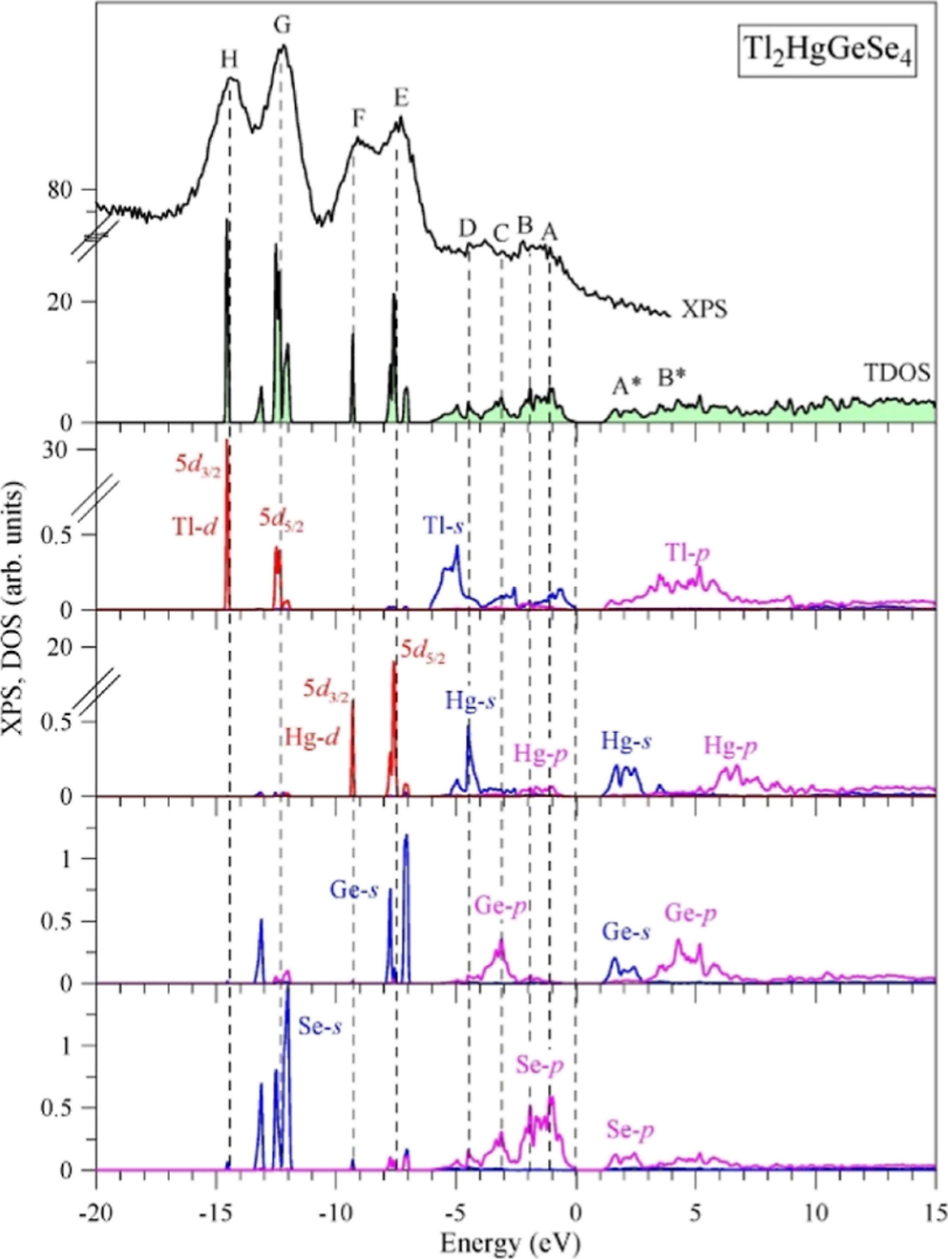
Comparison of the TDOS and main PDOS curves as calculated within the TB-mBJ + *U* + SO model and the experimental XPS spectrum of Tl_2_HgGeSe_4_.

The best agreement is achieved when using in the calculations the TB-mBJ functional for the XC potential, and we also involve the Hubbard amendment parameter *U* and the SO coupling effect (TB-mBJ + *U* + SO model). As can be seen from Figure 7, where comparison of the TDOS and main partial DOS (PDOS) curves as computing within the TB-mBJ + *U* + SO model and the experimental XPS spectrum of Tl_2_HgGeSe_4_ is presented, in such a case, we detect the good conformity of the energy locations of the experimental features E and F with the theoretical Hg 5d_5/2_ and Hg 5d_3/2_ sub-bands, as well as of the experimental spectral peculiarities G and H with the theoretical Tl 5d_5/2_ and Tl 5d_3/2_ sub-bands (it is worth mentioning that in the present GGA-PBE + *U* as well as TB-mBJ + *U* + SO calculations of Tl_2_HgGeSe_4_, we employed the values 0.4 Ry of the Hubbard correction parameters *U*_Tl_ and *U*_Hg_ for strongly correlated Tl 5d and Hg 5d electrons). It is worth indicating that we have gained the above Hubbard parameters *U*_Tl_ and *U*_Hg_ to be equal to 0.4 Ry for strongly correlated Tl 5d and Hg 5d electrons only as the adjusting parameters since they are very difficult to be retrieved theoretically because of rather complicated band structure DFT calculations for the Tl_2_HgGeSe_4_ compound. Employing this mBJ + *U* + SO technique, we have obtained the energy band gap being in fair agreement with the experimental measurements of *E*_g_, and we also reached an excellent correspondence of peculiar features and their energy locations for the theoretical curve of total DOS and the experimental XPS spectrum recorded in the valence band region. We have involved the consideration of the Hubbard correction parameters *U* only for Tl 5d and Hg 5d states because of their location being near the Tl_2_HgGeSe_4_ valence band bottom (Figure 7), while Se 3d and Ge 3d states are positioned far away from the valence band bottom, in fact with binding energies of about 53.4 and 30.3 eV, respectively, as the experimental data listed in Table 3 present. Following the above arguments, we did not treat the Hubbard parameter *U* for Se 3d and Ge 3d states when calculating the electronic structure of Tl_2_HgGeSe_4_ within the mBJ + *U* + SO technique The TB-mBJ + *U* + SO technique also gives SO couplings to be equal to about 2.1 eV and 1.7 for Tl 5d_5/2,3/2_ and Hg 5d_5/2,3/2_ electronic states, respectively. The above theoretical values are in excellent conformity for the corresponding experimental SO coupling magnitudes of those electronic states, as the data listed in Table 3 and the XPS measurements plotted in Figure 7 indicate.

**Figure 8:**
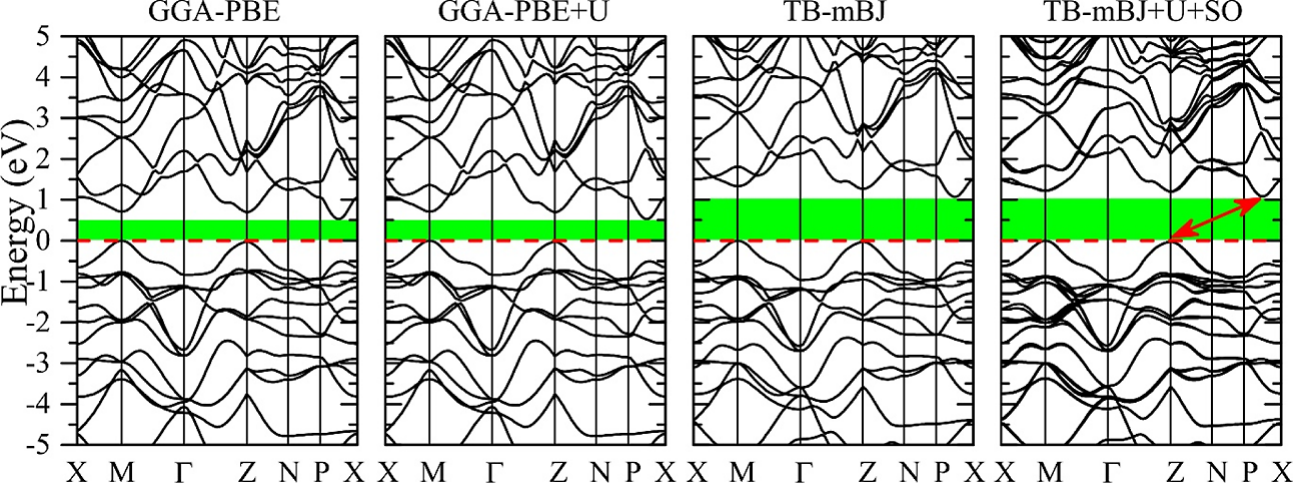
Band dispersions as calculating through the paths defined by points of high symmetry of the Tl_2_HgGeSe_4_ crystal: calculations within GGA-PBE, GGA-PBE + *U*, TB-mBJ, and TB-mBJ + *U* + SO models.

The results of DFT calculations of band dispersions of Tl_2_HgGeSe_4_ are presented in Figure 8. These data bring to the statement that the Tl_2_HgGeSe_4_ crystal is a nondirect band gap semiconductor because in this quaternary selenide, the maximum of the valence band is noticed in the high-symmetry point *Z*, while the minimum of the conduction band is detected at point k located along the direction determined by the high-symmetry points *P* and *X*. These theoretical results present that in the Tl_2_HgGeSe_4_ crystal, the energy band gap nature is different from that of the related Tl_2_CdD^IV^Se_4_ (D^IV^ = Ge and Sn) and Tl_2_HgSnSe_4_ compounds, which, according to the theoretical band structure calculations and experimental measurements reported in refs (50), (52), and (73), are direct band gap semiconductors. Following the data of the present DFT computation listed in Table 4, the *E*_g_ value of Tl_2_HgGeSe_4_ as determined based on the GGA-PBE approach^59^ is about 0.54 eV smaller in comparison with that based on the TB-mBJ approach.^60^ The inclusion of the Hubbard amendment parameter *U* in the calculation process does not affect much the theoretical energy band gap magnitude of the Tl_2_HgGeSe_4_ crystal (Table 5).

**Table 5:** Energy Band Gap Values, *E*_g_, of Tl_2_HgGeSe_4_ Calculated by Different Models

model used in the calculations	*E*_g_, eV
GGA	0.560
GGA + *U*	0.576
MBJ	1.105
MBJ + *U* + SO	1.114

**Figure 9:**
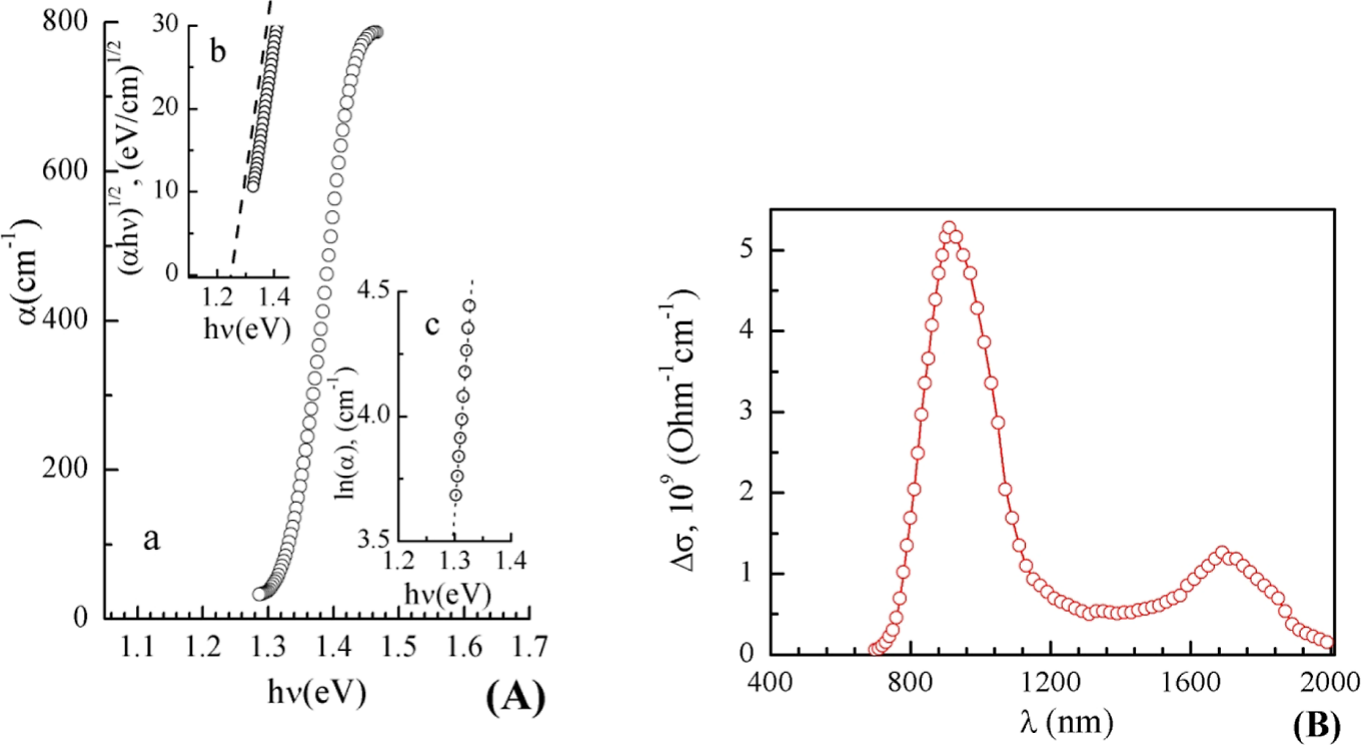
(A) Spectral distribution of the absorption coefficient of the Tl_2_HgGeSe_4_ crystal as measured at 300 K and (B) spectral distribution of the photoconductivity of the crystal at 100 K.

To verify these theoretical data, we have performed experimental estimations of the energy band gap of the Tl_2_HgGeSe_4_ crystal. Figure 9A presents a study of the spectral distribution of the absorption coefficient α (panel a) at the edge of the fundamental absorption region as measured at *T* = 300 K. The energy band gap, *E*_g_, of the crystal under consideration was estimated by the Tauc method: the dependence of (α*h*ν)^1/2^ upon photon energy *h*ν is shown in Figure 9A (panel b). The experimentally determined indirect energy band gap is equal to 1.28 eV at room temperature. This experimental value for the Tl_2_HgGeSe_4_ crystal is in a reasonable correspondence to *E*_g_ = 1.114 eV as derived in the present TB-mBJ + *U* + SO calculations (Table 5).

Below the region of strong absorption, an exponential dependence of α upon photon energy *h*ν follows (Figure 9A, panel c), which indicates the fulfillment of Urbach’s rule. From the experimental results presented in Figure 9A (panel c), the Urbach energy (*E*_U_ = Δ(*h*ν)/Δ(lnα)) was determined: it is equal to 32 meV in the Tl_2_HgGeSe_4_ crystal. Such values of the Urbach energy are typical for multicomponent semiconductors.^77,78^ Measurements of the sign of thermo-EMF (∼10 μV/K) indicate that the Tl_2_HgGeSe_4_ crystal is a high-resistance semiconductor with p-type electroconductivity and a specific electrical conductivity of σ ∼ 10^–8^ Ω^–1^ cm^–1^ (at = 300 K). Regarding possible origins of the exponential tail in the spectral distribution of the absorption coefficient, in the literature, several mechanisms were suggested to be characteristic, in particular, fluctuations in bond angles and lengths, electronic transitions between localized states in the tails of the band edges, etc. It is believed that the density of such states decreases exponentially with the photon energy.^79^ The exponential increase in the absorption coefficient in the region of the absorption edge could be explained by the transitions involving tails of densities of states in the valence band and in the conduction band.^79^ The shape and size of these tails depend on the presence of different types of disordering. For example, for perfect CdS single crystals, the Urbach energy is equal to 0.02 eV, while for CdS glass, it is about 0.1–0.2 eV.^80^ In the crystal under study, when the photon energy decreases (λ > 990 nm), the Urbach region passes into the region of slowly decreasing weak absorption (“weak absorption tail”), which is due to the peculiarities of the structure of the energy bands of amorphous or defect semiconductors.^81^ At λ > 1030 nm, the residual absorption in Tl_2_HgGeSe_4_ is ∼0.8 cm^–1^.

The Tl_2_HgGeSe_4_ crystal, as the present data show, is a photosensitive semiconductor. Measurements of the spectral dependence of the photoconductivity of Tl_2_HgGeSe_4_ (Figure 9B) reveal the presence of two photoconductivity maxima in this crystal. The first maximum positioned at λ ≈ 910 nm lies in the region of the intrinsic absorption band and corresponds to the energy of 1.36 eV at *T* = 100 K, which coincides well with the band gap estimated from the spectral dependence of the absorption coefficient (1.28 eV at *T* = 300 K). The maximum of impurity photoconductivity (λ ≈ 1730 nm) originates due to the ionization of impurity centers. According to the position of the impurity photoconductivity maximum (Figure 9B), the ionization energy of the impurity center was estimated to be *E*_a_ = *E*_v_ + (0.56 ± 0.02) eV. With increasing temperature, we observe a temperature quenching of the photosensitivity of the studied crystal. This temperature quenching is most likely associated with the increasing efficiency of the recombination flow through defect centers.

**Figure 10:**
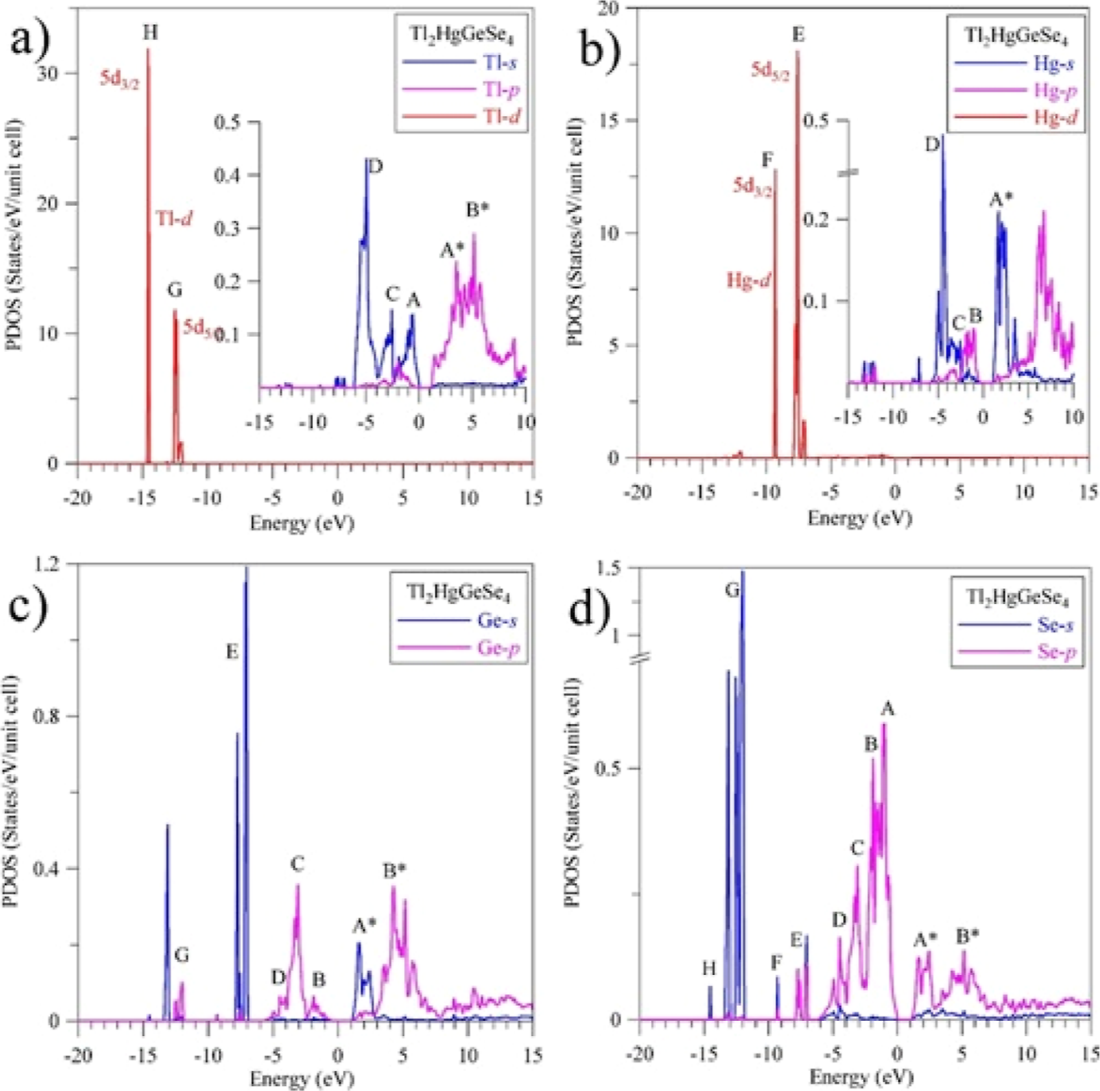
Detailed partial densities of states of the Tl_2_HgGeSe_4_ crystal calculated within the TB-mBJ + *U* + SO model: (a) Tl, (b) Hg, (c) Ge, and (d) Se.

Following the fact that the finest correspondence with the experiment is detected when performing DFT calculation using the TB-mBJ + *U* + SO model, in Figure 10, we present detailed theoretical data concerning features of filling the main section of the valence band spreading from 0 until −6.1 eV by electronic states of peculiar symmetries associated with the constituting atoms of the Tl_2_HgGeSe_4_ crystal. These data bring to the statement that near the valence band topmost of Tl_2_HgGeSe_4_, the principal contributions give Se 4p states, with less contribution of Tl 6s states, too. The upper sub-band A of the valence band of Tl_2_HgGeSe_4_ centered at −1.1 eV has taken shape by Se 4p states and, with less contribution, by Tl 6s and Hg 6p states, whereas somewhat lower sub-band B centered at −1.9 eV is dominated by Se 4p states, with substantially less contribution of Ge 4p, Hg 6p, and Tl 6s, 6p states as well. The central sub-band C is predominated by the contribution of Ge 4p states, with much fewer contribution of Se 4p and Tl 6s states. The lower sub-band D centered at −4.6 eV prevailed from contribution of Hg 6s states, while the valence band bottom of Tl_2_HgGeSe_4_ is dominated by Tl 6s states.

From Figure 10, it can be noted that the conduction band bottom (peculiarity A*) of the Tl_2_HgGeSe_4_ crystal takes shape mainly by unoccupied Ge 4p states, with less contribution of unoccupied Ge 4s, Se 4p, and Tl 6p states, too. The upper sub-band B* prevails by unoccupied Tl 6p and Ge 4p states, while above this sub-band, the contribution of unoccupied Hg 6p states prevails (Figure 10).

Figure 10 displays that the valence band of the Tl_2_HgGeSe_4_ crystal is characterized by essential hybridization of Se 4p states with Hg 6p and Tl 6p states in the upper area, with Ge 4p states in the central area, and with Hg 6s and Tl 6s states in the lower area of the valence band. The presence of this essential hybridization degree of the above-mentioned PDOS causes the existence of a significant covalent component (furthermore to ionic component) in the total combination of chemical bonding in the Tl_2_HgGeSe_4_ crystal, being in fair correspondence with the conclusion retrieved based on XPS evaluations of the binding energies listed in Table 3. Such a feature of the chemical bonding of the Tl_2_HgGeSe_4_ crystal is similar to that established earlier for the related Tl_2_HgSnSe_4_ and Tl_2_CdD^IV^Se_4_ (D^IV^ = Ge and Sn) compounds.^50,52,73^

**Figure 11:**
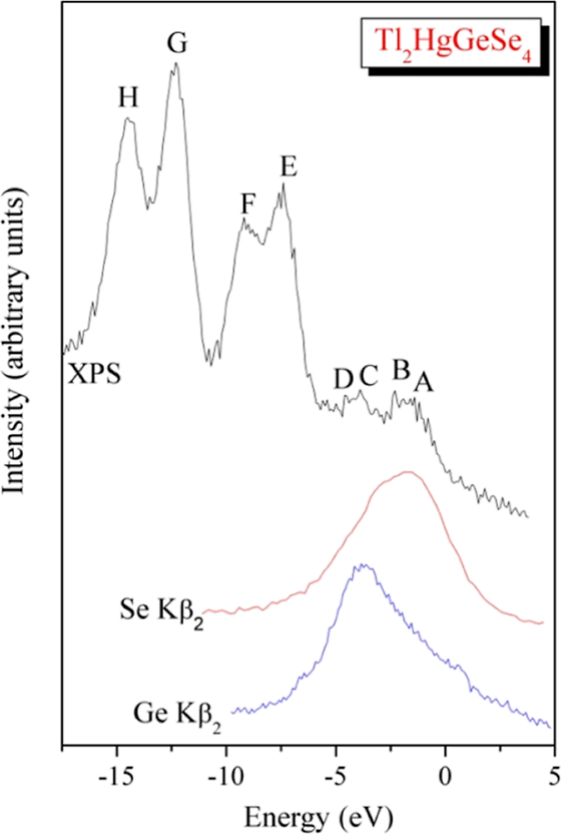
XPS spectrum measured in the area of the valence band of the Tl_2_HgGeS_4_ crystal compared on a common energy scale with its XES Se(Ge) Kβ_2_ bands.

To examine the results of the present DFT suggestions concerning the principal energy locations of 4p states related to Se and Ge atoms in the valence band of the Tl_2_HgGeSe_4_ crystal, we have measured the X-ray emission spectra bringing knowledge on these states, the Se Kβ_2_ and Ge Kβ_2_ XES bands, respectively. Results of comparison of these XES bands and the XPS spectrum of the untreated Tl_2_HgGeSe_4_ crystal recorded in the valence band region are shown in Figure 11. For this comparison, we employ the usually used technique of the XPS and XES spectra reported elsewhere.^82,83^ As could be noted by comparing the data shown in Figures 10 and 11, we detect good correspondence of the theory and experiment regarding the principal energy locations of 4p states related to Se and Ge atoms in the valence band of the Tl_2_HgGeSe_4_ crystal. In particular, the maximum of the Se Kβ_2_ XES band is located in the vicinities of theoretical features A and B, being in fair correspondence with the theoretical Se 4p PDOS curve (Figure 10d). Further, the XES experiments feature that essential contribution of Se 4p states should take place in the central and lower parts of the Tl_2_HgGeSe_4_ crystal valence band (Figure 11) being in agreement with the theoretical indications presented in Figure 10d. Furthermore, the main maximum of the Ge Kβ_2_ XES band is located in the vicinities of the theoretical feature C. Therefore, the main contribution of Ge 4p states is experimentally detected in the central area, with some contributions of these electronic states in the upper part of the valence band of the Tl_2_HgGeSe_4_ crystal, again confirming the theoretical suggestions for the location of Ge 4p electronic states (Figure 10c). Similar features of filling the valence band ranges by electronic 4p states associated with selenium and germanium atoms were detected by XES measurements and/or first-principles computations of other related Ge-bearing selenides, in fact Tl_2_CdGeSe_4_^51,52^ and Cu_2_HgGeSe_4_.^71^ It should be noted that, in accordance with the theoretical computing data plotted in Figure 10, contribution of electronic s-like states associated with mercury and thallium is expected to be also essential in the lower part of the valence band of Tl_2_HgGeSe_4_. Nevertheless, our existent facilities do not allow verifying experimentally these theoretical suggestions for the crystal under study.

### 4.3 Main Optical Properties of the Tl_2_HgGeSe_4_ Crystal as Evidenced from the DFT Computation

**Figure 12:**
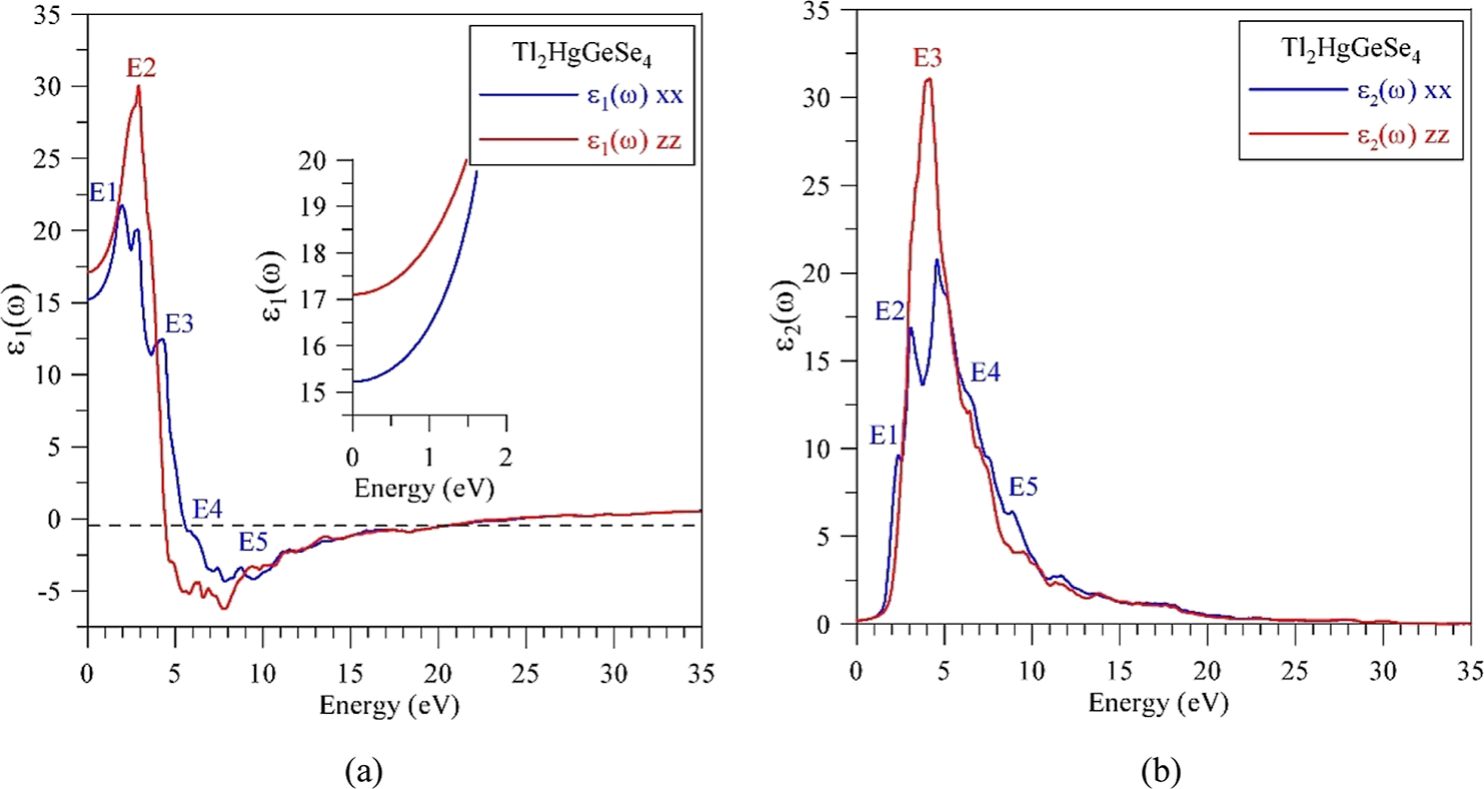
Computational models of (a) real ε_1_ (ω) and (b) imaginary ε_2_ (ω) components of the dielectric function of the Tl_2_HgGeSe_4_ crystal.

The real ε_1_ (ω) and imaginary ε_2_ (ω) components of the dielectric function of Tl_2_HgGeSe_4_ derived theoretically within the TB-mBJ + *U* + SO model are plotted in Figure 12. The present data imply that the theoretical static dielectric constants, i.e., those determined at zero frequency, for the real component of the dielectric function were found to be equal to ε_1_^*xx*^(0) = 15.2262 and ε_1_^*zz*^(0) = 17.1043, bringing to the conclusion that the average static real component of the dielectric function is ε_1_^average^(0) = 16.1653. The above-mentioned values are somewhat higher in comparison with those determined to be characteristic of the Cd-bearing counterpart, Tl_2_CdGeSe_4_, where they are following: ε_1_^*xx*^(0) = 12.1388 and ε_1_^*zz*^(0) = 13.2692.^52^

Figure 12a reveals that beginning from the static values, the ε_1_ (ω) function presents fast enhancement until reaching the maxima E1 (1.78 eV) and E2 (2.9 eV), followed by its sharp decreasing until the minimum located at nearly 8 eV. With further increasing photon energies, the ε_1_ (ω) function increases its intensity. As can be noticed from Figure 12a, in addition to the maxima E1 and E2, formations of peculiarities E3 (4.3 eV), E4 (∼6.3 eV), and E5 (∼8.8 eV) are distinguished for the real constituent of the dielectric function of Tl_2_HgGeSe_4_. The biggest values of the ε_1_ (ω) function in this crystal are observed at nearly 1.7–3.9 eV; this range covers totally the whole visible light region and a part of near-UV area. The real component of the dielectric function of Tl_2_HgGeSe_4_ crosses zero first time in the interval 4.2–5.2 eV and the second time at around 24 eV. This fact features that, in the mentioned energy area being in the range from mid-UV up to extreme UV, the Tl_2_HgGeSe_4_ crystal reveals metallic properties with respect to the interaction with EM waves. The imaginary component ε_2_ (ω) of the dielectric function of Tl_2_HgGeSe_4_ is distinguished by the existence of sharp increase beginning from about 1.1 eV until the maximum E3 located nearly 4.1 eV and, with enhancing photon energies, it goes down to near-zero values for energies nearly 35 eV. In addition to the maximum E3, the ε_2_ (ω) function reveals peculiarities E1 (2.38 eV), E2 (∼3.1 eV), E4 (∼6.5 eV), and E5 (∼8.9 eV). The biggest magnitudes of imaginary constituent of the dielectric function of Tl_2_HgGeSe_4_ are detected for photon energies from 2.9 until 5.9 eV that comprise the area from violet color of visible light up to mid-UV. As could be noticed from Figure 12, the real ε_1_ (ω) and imaginary ε_2_ (ω) constituents of the dielectric function of Tl_2_HgGeSe_4_ reveal high anisotropy degree in the energy area from 1.5 up to about 10 eV, mainly in the vicinities of the maxima/peculiarities E1–E5.

**Figure 13:**
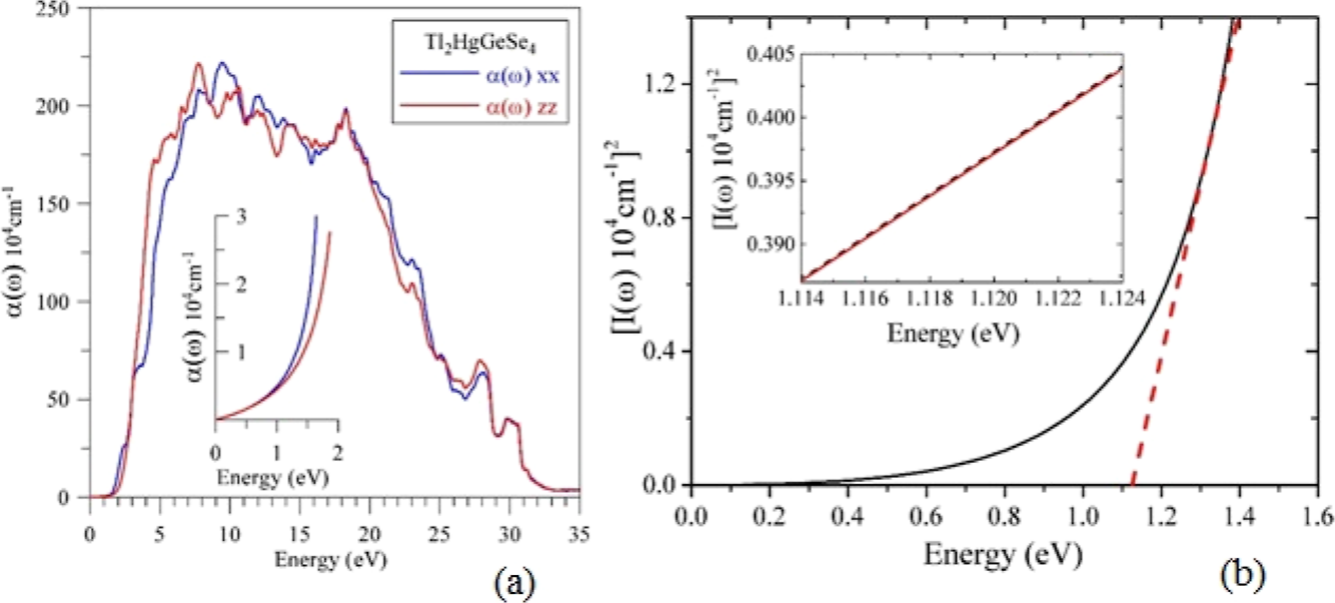
Computational (a) absorption coefficient α(ω) and (b) (*I*(ω)10^4^ cm^–1^)^2^ dependence in the Tl_2_HgGeSe_4_ crystal.

Theoretically retrieved optical coefficient of absorption α(ω) of Tl_2_HgGeSe_4_ is shown in Figure 13a. It is apparent that the α(ω) function goes abruptly up beginning from the first determinative point, which is located at a photon energy of nearly 1.114 eV (Figure 13b). In a comparatively broad energy region, at least from 3.7 until about 24 eV covering area from near-UV until extreme UV, the α(ω) spectrum possesses values above 10^6^ cm^–1^ that allows for the statement about a good prospective for the Tl_2_HgGeSe_4_ crystals to be effectively used in optoelectronics.

**Figure 14:**
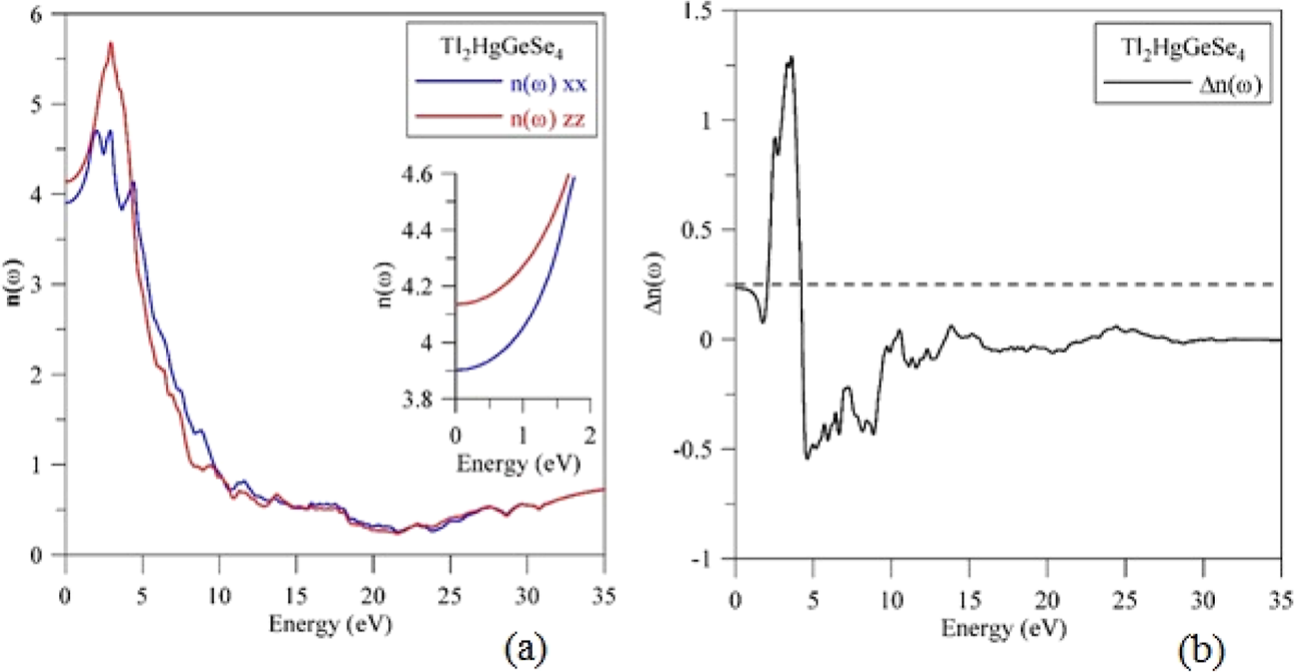
Computational (a) refractive index *n*(ω) and (b) birefringence Δ*n*(ω) of the Tl_2_HgGeSe_4_ crystal.

Figure 14a displays the refractive index *n*(ω) of Tl_2_HgGeSe_4_, whereas the birefringence Δ*n* of this crystal is given in Figure 14b. The present calculating results feature that the shapes and energy distributions of peculiar features of the *n*(ω) and ε_1_ (ω) spectra of the Tl_2_HgGeSe_4_ crystal are rather similar (cf. Figures 12a and 14a). The static components of the *n*(ω) function of Tl_2_HgGeSe_4_ are determined to be the following: *n*^*xx*^(ω) = 3.9022 and *n*^*zz*^(0) = 4.1358. Again, these static magnitudes of the refractive index of Tl_2_HgGeSe_4_ are somewhat higher in comparison with those of related Cd-bearing selenide Tl_2_CdGeSe_4_ (*n*^*xx*^(0) = 3.4841 and *n*^*zz*^(0) = 3.6427).^52^ It is well known that the birefringence Δ*n*(ω) of a material is defined by the difference of the refraction indexes related to extraordinary and ordinary rays, *n*_e_(ω) and *n*_0_(ω), respectively, and the Δ*n*(ω) function peculiarities are worthy to be analyzed mainly for photon energies that do not exceed the energy band gap value, *E*_g_, i.e., in the lack of absorption. The birefringence Δ*n*(ω) of the Tl_2_HgGeSe_4_ crystal is characterized by the static value Δ*n*(0) = 0.2336, and in the interval 0–1.114 eV, it features positive magnitudes. This result implies that the polarization of the fast EM waves in the Tl_2_HgGeSe_4_ crystal is perpendicular to its optical axis. The birefringence Δ*n*(ω) of the Tl_2_HgGeSe_4_ crystal is positive in the energy range from 0 to about 4 eV, followed by negative values with increasing photon energy up to near 11 eV and its oscillating near zero with a further increase of photon energies up to 35 eV.

**Figure 15:**
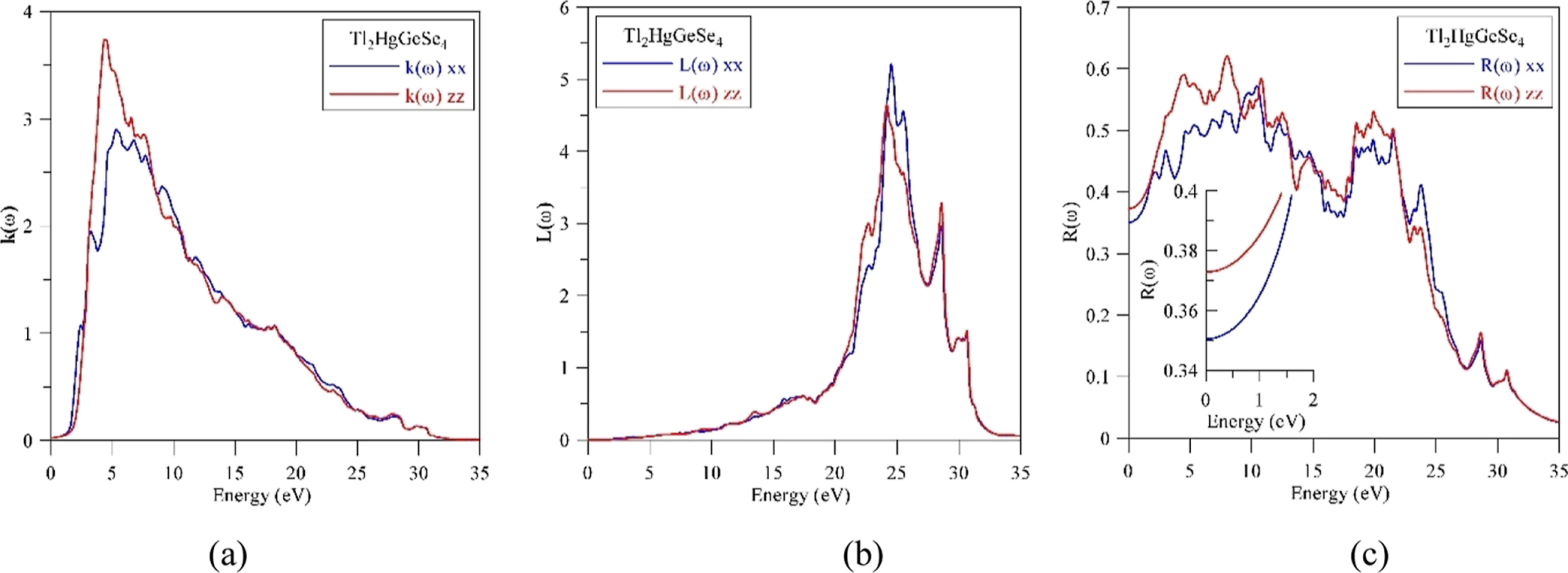
Computational (a) extinction coefficient *k*(ω), (b) electron energy loss spectrum *L*(ω), and (c) optical reflectivity *R*(ω) of the Tl_2_HgGeSe_4_ crystal.

The extinction coefficient *k*(ω) of Tl_2_HgGeSe_4_ plotted in Figure 15a resembles the spectrum of the imaginary component ε_2_ (ω) of the dielectric function, while the spectrum of electron energy loss *L*(ω), as Figure 15b presents, reveals its maximum values at around 25 eV. This value defines the plasmon energy in the Tl_2_HgGeSe_4_ crystal. The coefficient of optical reflectivity *R*(ω) of the crystal under study is presented in Figure 15c. These theoretical data show that static *R*(ω) magnitudes are defined to be the following: *R*^*xx*^(0) = 35.0496 and *R*^*zz*^(0) = 37.2818%. These values are also bigger as compared to those of the related selenide Tl_2_CdGeSe_4_ where they are as follows: *R*^*xx*^(0) = 30.6905% and *R*^*zz*^(0) = 33.402%.^52^ Dispersion of the reflectivity coefficient *R*(ω) of Tl_2_HgGeSe_4_ is distinguished by the existence of two bands: one broader and more intensive centered at about 8 eV and the second one being much narrower and slightly less intensive centered at about 20 eV.

It should be indicating that our DFT first-principles computational data reveal that the edge of optical absorption of the Tl_2_HgGeSe_4_ crystal appears at 1.114 eV. This value of photon energy corresponds to the infrared spectral range and, in accordance to the theoretical data presented in Figure 13, the α(ω) spectrum possesses high magnitudes (bigger than 10^6^ cm^–1^) in a comparatively wide energy range, at least from 3.7 until about 24 eV covering area from near-UV until extreme UV. The highest values of the ε_1_ (ω) function in the crystal under study are theoretically detected in the 1.7–3.9 eV range covering the whole visible light region and near-UV spectrum. The biggest values of the imaginary constituent of dielectric function, ε_2_ (ω), of Tl_2_HgGeSe_4_ are observed being in the 2.9–5.9 eV area that covers the spectral region from violet color of visible light up to mid-UV. The shapes and energy locations of peculiar features of the real and imaginary components of dielectric function, ε_1_ (ω) and ε_2_ (ω), of Tl_2_HgGeSe_4_ resemble those of the refractive index *n*(ω) and the extinction coefficient *k*(ω), respectively. The above theoretical results feature that all the computational optical constants are characterized by rather pronounced nonisotropic behaviors of their two constituents of the second rank tensor generally near the maxima/extrema/peculiarities positioned in the energy area up to 10 eV. The above-mentioned peculiar features of the optical constants of Tl_2_HgGeSe_4_ suggest that the crystal could be effectively used in optoelectronics. These peculiarities for the calculating optical coefficients of the Tl_2_HgGeSe_4_ crystal satisfy specific requirements of photocatalysts for water splittings.^84^ Additionally, the present computing results reveal the existence of an energy band gap of 1.114 eV, whereas the experimental measurements give *E*_g_ = 1.28 eV for the Tl_2_HgGeSe_4_ crystal. These *E*_g_ values are within the region for materials that could be applied as thin-film absorbers of solar cell technology.^11,85^ In addition, the Tl_2_HgGeSe_4_ crystal features p-type electroconductivity that is very important for materials used in thin-film solar cells. Furthermore, the Tl_2_HgGeSe_4_ crystal structure of the tetragonal *I*4̅2*m* space group is noncentrosymmetric. Therefore, one could expect some possible applications of Tl_2_HgGeSe_4_ as a material for nonlinear optical devices. However, the verification of the above suggestion requires specific experimental efforts in future research.

### 4.4 Raman Spectra

**Figure 16:**
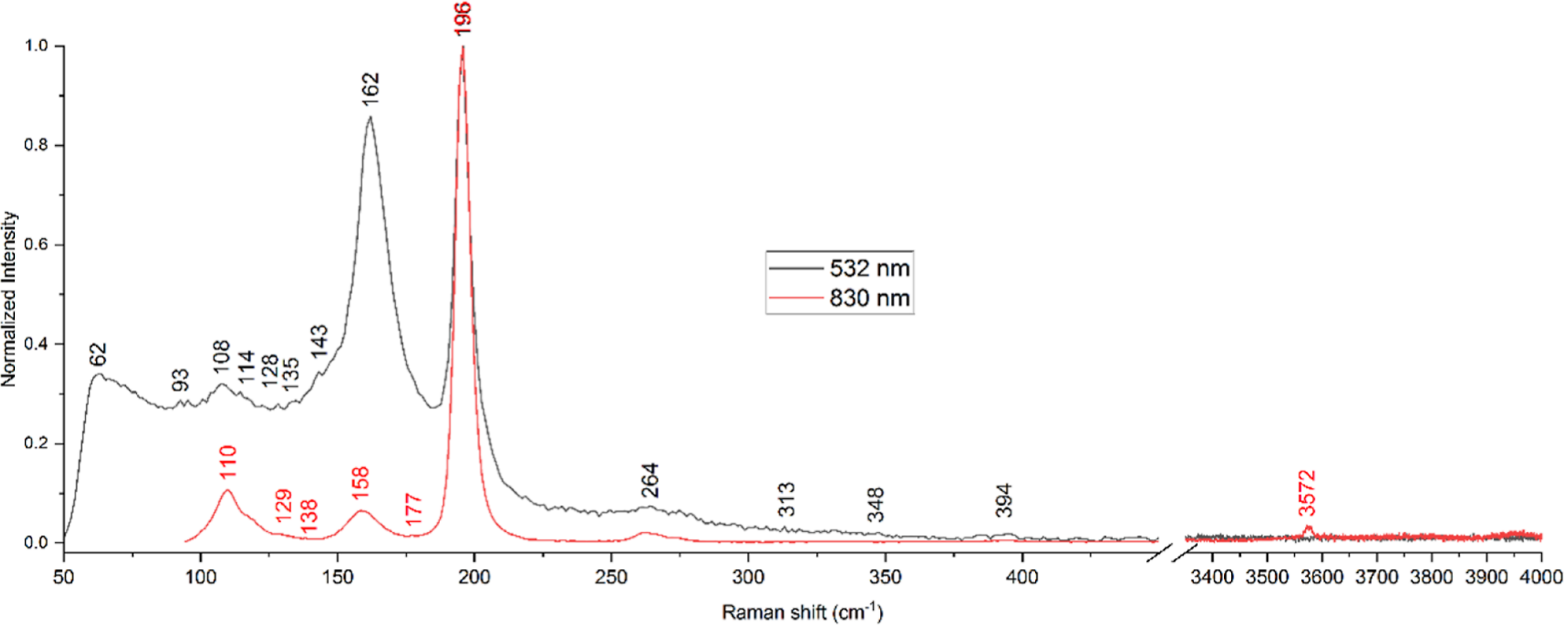
Raman spectra of the Tl_2_HgGeSe_4_ crystal derived using laser excitations at 532 (black) and 830 nm (red curves).

The Raman spectra as recorded for the Tl_2_HgGeSe_4_ crystal using laser excitations at 532 and 830 nm are presented in Figure 16. It should be mentioned that, in the literature, there is a lack of data concerning the vibrational spectra of the studied compound. The measurements using 532 and 830 nm excitation wavelengths give similar results (Figure 16): the highest peak at 196 cm^–1^ and some smaller by intensity peaks around 110, 160, and 264 cm^–1^. In the structure of Tl_2_HgGeSe_4_, the stretching Hg–Se, Tl–Se, and Ge–Se modes should be present as it forms a quasi-ternary system Tl_2_Se–HgSe–GeSe_2_.^46^

The maximum at 196 cm^–1^ of the Raman spectra presented in Figure 16 can be attributed to the symmetric stretching mode of α-GeSe_2_.^86,87^ The peak around 264 cm^–1^ could be assigned to the asymmetric stretching vibration of GeSe_4/2_ tetrahedral units,^86,88^ the vibration around 162 cm^–1^ as vibrations of GeSe_2_,^86^ and the peak around 93 cm^–1^ as TO of the GeSe_2_ crystal mode.^86^ The reference results exist for the glass; nevertheless, the IR peak values obtained for the Ge_*y*_Se_1–*y*_ glass and Raman modes of crystalline GeSe_2_ are similar.^86^

Vibrations of Tl_2_Se should be present around 310 cm^–1^, but no direct evidence of their presence was found for the studied sample.^88^ However, there is a very small peak at 313 cm^–1^ (comparable with a noise level), which can be associated with a quite small concentration of Tl_2_Se as it was reported^88^ that the addition of 20–30 mol % of Tl_2_Se should cause the disappearance of the peak at 264 cm^–1^ and increase of peak at 310 cm^–1^ correlated with the formation of GeSe_3/2_Se–Tl^+^; therefore, there is a possibility that the small-intensity peak at 310 cm^–1^ superimposes the peak at 264 cm^–1^. On the other hand, the vibrations of the crystal of TlSe^89^ should be present in the range around 134 cm^–1^ (TO_1_), 179 cm^–1^ (LO_1_) or 158 cm^–1^ (TO_1_), 175 cm^–1^ (LO_1_), 88 cm^–1^ (TO_2_), and 108 cm^–1^ (LO_2_) for A_2u_ or E_u_ symmetry types, respectively.

Furthermore, the vibrations of HgSe^90^ should be present as strong peaks at 135 cm^–1^ (TO_1_ of HgSe), shoulders at 173 cm^–1^ (LO_1_ of HgSe) and broad peaks around 345 cm^–1^. The measurement confirms its presence as there are small peaks at 135 and 348 cm^–1^ detectable for the Raman spectrum obtained with excitation by the 532 nm laser. The Hg–Hg stretching (A_1g_ mode) vibration of Hg (I) in halides should be found below 186 cm^–1^ (the frequencies of the peak decrease from the lightest fluoride (around 186 cm^–1^) to the heaviest iodide (around 113 cm^–1^)).^91^ On the other hand, the mercury(II) halides should give a peak around 320 cm^–1^.^92^

The vibration at 3572 cm^–1^ observed using 830 nm excitation wavelength can be interpreted as the vibrations of the hydroxyl group.^93^ The small intensity of the peak can be explained as an interaction of selenium atoms (of the surface layer) with the air, not as a hydroxyl group being a part of the crystal structure of the studied sample as selenium has a great tendency to react with the hydroxyl group.^94^

In summary, it should be mentioned that all the theoretical results reported in the present work were derived by assuming the crystal structure of the Tl_2_HgGeSe_4_ crystal belonging to noncentrosymmetric SG *I*4̅2*m*. However, as the data listed in the Supporting Information reveal, the structure of Tl_2_HgGeSe_4_ can also be presented in centrosymmetric SG *I*4/*mcm*. The problem of determination of the centrosymmetric/noncentrosymmetric crystal structure belonging to the A_2_B^II^D^IV^X_4_-type compounds (A = Cu and Tl; B^II^ = Zn, Cd, and Hg; D^IV^ = Si, Ge, and Sn; and X = S, Se, and Te) was known long ago. There exist a number of publications where a noncentrosymmetric SG *I*4̅2*m* of the structure is accepted for these compounds.^95−100^ Previously, this space group was already attributed to the compound Tl_2_HgGeSe_4_ based on the powder XRD pattern of the Tl_2_HgGeSe_4_ alloy.^46^ In the present work, we have performed structure refinement for the Tl_2_HgGeSe_4_ crystal aiming only to identify that the crystal is a single-phase material with the crystal structure belonging to Tl_2_CdGeTe_4_ type (SG *I*4®2*m*) as it is suggested previously for many other A_2_B^II^D^IV^X_4_-type quaternary chalcogenides,^94−100^ including Tl_2_HgGeSe_4_.^46^ Assuming the structure belonging to SG *I*4̅2*m*, we have performed DFT calculations of the electronic structure and optical properties of Tl_2_HgGeSe_4_, and the theoretical results are found to be in excellent agreement with the experimental measurements carried out for the Tl_2_HgGeSe_4_ crystal. Certainly, the possibility of a centrosymmetric crystal structure belonging to SG *I*4/*mcm* should be verified for Tl_2_HgGeSe_4_. The present problem with strict identification of the crystal structure of Tl_2_HgGeSe_4_ based on the powder XRD measurements can be partly caused by the fact that Tl and Hg are neighboring elements of the periodic table. Previously, the presence of two neighboring heavy chemical elements, Hg and Tl, was found to be the reason for impossibility of unequivocal identification of the crystal structure (centrosymmetric/noncentrosymmetric) of Tl_4_HgX_6_ halides (X = Br and I).^101,102^ The aforementioned information brings to the statement that additional attempts should be made in the future for Tl_2_HgGeSe_4_ with respect to the possibility of its crystallization within centrosymmetric SG *I*4/*mcm*.

## 5 Conclusions

We report the results of a complex investigation of the electronic and optical properties of a Tl_2_HgGeSe_4_ crystal, which was performed by employing the Bridgman–Stockbarger growth technique, for the first time, in this work. It is obvious that the sample is not a single crystal and most likely has a layered structure. The observed play of color on the surface (iridescence and interference) is due to the manifestation of layers of different thicknesses. Such crystals are usually characterized by pronounced cleavage. In such cases, problems are expected with obtaining bulk crystals and with their mechanical processing (cutting and polishing), manufacturing optical elements of the required geometry, and crystallographic orientation when they are used in optics, in particular, nonlinear. Therefore, future attempts are necessary to obtain bulk centimeter-sized single crystals. However, the present XPS measurements reveal that the Tl_2_HgGeSe_4_ crystal surface possesses rather small moisture sensitivity and the 3 kV Ar^+^ ion-beam-induced treatment evokes decreasing of the mercury content in the top near-surface analyzing layer. In accordance with XPS evaluation of the binding energies for core-level electrons of the composing atoms, the Tl_2_HgGeSe_4_ crystal should possess a substantial covalent constituent in the system of total chemical bonding. This experimental finding is verified by the theoretical first-principles DFT computational results, which indicate that the mentioned essential covalent component of the chemical bonding is provided by hybridization of Se 4p states with Hg 6p and Tl 6p states in the upper section, with Ge 4p states in the central part, and with Hg 6s and Tl 6s states in the lower area of the valence band of Tl_2_HgGeSe_4_. The present theoretical data indicate that for the finest agreement of theory and experiments, the band structure calculations of the Tl_2_HgGeSe_4_ crystal should be made employing the TB-mBJ + *U* + SO model. In accordance with the TB-mBJ + *U* + SO calculations, Se 4p states provide the principal contributions at the topmost and in the upper and central portions of the valence band of Tl_2_HgGeSe_4_. The central part of the valence band prevailed by contributions of Ge 4p states, and its lower part prevailed by contribution of Hg 6s states, while the valence band bottom of Tl_2_HgGeSe_4_ prevailed by contribution of Tl 6s states. Further, the conduction band bottom of the Tl_2_HgGeSe_4_ crystal takes shape mainly by unoccupied Ge 4p states, with lesser contributions of unoccupied Ge 4s, Se 4p, and Tl 6p states, too. The present DFT computing results reveal that the Tl_2_HgGeSe_4_ crystal is a nondirect semiconductor: the maximum of the valence band is noticed in the high-symmetry point *Z*, while the minimum of the conduction band is detected at point k positioned along the direction determined by high-symmetry points P and X. The present experimental measurements feature that the Tl_2_HgGeSe_4_ crystal is characterized by p-type electroconductivity possessing an indirect energy band gap of 1.28 eV. The Urbach energy (*E*_U_ = Δ(*h*ν)/Δ(lnα)) was determined to be equal to 32 meV in the Tl_2_HgGeSe_4_ crystal. The Tl_2_HgGeSe_4_ crystal is a high-resistance semiconductor with a specific electrical conductivity of σ ∼ 10^–8^ Ω^–1^ cm^–1^ (at = 300 K). The Tl_2_HgGeSe_4_ crystal is a photosensitive semiconductor. Measurements of the spectral dependence of the photoconductivity of Tl_2_HgGeSe_4_ reveal the presence of two photoconductivity maxima in this crystal. The first maximum positioned at λ ≈ 910 nm lies in the region of the intrinsic absorption band and corresponds to an energy of 1.36 eV at *T* = 100 K, which coincides well with the band gap estimated from the spectral dependence of the absorption coefficient (1.28 eV at *T* = 300 K). The maximum of impurity photoconductivity (λ ≈ 1730 nm) originates due to the ionization of impurity centers. According to the position of the impurity photoconductivity maximum, the ionization energy of the impurity center was estimated to be *E*_a_ = *E*_v_ + (0.56 ± 0.02) eV.

Our calculating results allow for the statement that the absorption coefficient α(ω) possesses magnitudes bigger than 10^6^ cm^–1^ in a comparatively wide energy area, at least from 3.7 until about 24 eV covering area from near-UV until extreme UV. Further, the real constituent of the dielectric function, ε_1_ (ω), possesses the highest values in the 1.7–3.9 eV range covering the total visible light region and the near-UV spectrum, while its imaginary component, ε_2_ (ω), reveals the biggest values in the 2.9–5.9 eV area that covers the spectral range from violet color of visible light up to mid-UV. The above theoretical results feature that all the computational optical constants are characterized by rather pronounced nonisotropic behaviors of their two components of the tensor of second rank generally in the vicinity of the maxima/extrema/peculiarities positioned in the energy region 0–10 eV. The measurements of the Raman spectra for the Tl_2_HgGeSe_4_ crystal using laser excitations at 532 and 830 nm allow for detecting a number of vibrations that could be assigned to different stretching Hg–Se, Tl–Se, and Ge–Se modes in this crystal. The present results allow for suggesting possible use of the Tl_2_HgGeSe_4_ crystal in optoelectronics and nonlinear optics as well as thin-film absorbers of solar cell technology.

## Supporting Information Available

Crystallographic data for Tl_2_HgGeSe_4_ (PDF)
