# Supplementary material for: Insights from Experiment and Theory on Peculiarities of the Electronic Structure and Optical Properties of the Tl2HgGeSe4 Crystal
Source: Inorg Chem. 2023 Oct 4;62(41):16691–709. doi: 10.1021/acs.inorgchem.3c01756 (PMC10583210; doi:10.1021/acs.inorgchem.3c01756)
Supplement: Supplementary file 1 — ic3c01756_si_001.pdf [file ic3c01756_si_001.pdf]

Supporting information

Insights from experiment and theory on peculiarities of the electronic structure and optical properties of the $Tl_2HgGeSe_4$ crystal

Tuan V. Vu [1,2], Oleg Khyzhun [3,4,*], Galyna L. Myronchuk [4], Mariana Denysyuk [4], Lyudmyla Piskach [5], Andrij O. Selezen [5], Ilona Radkowska [6], Anatolii O. Fedorchuk [7], Svitlana S. Petrovska [3], Vira A. Tkach [3], Michał Piasecki [6,8**]

[1]Laboratory for Computational Physics, Institute for Computational Science and Artificial Intelligence, Van Lang University, Ho Chi Minh City, Vietnam

[2]Faculty of Mechanical - Electrical and Computer Engineering, School of Technology, Van Lang University, Ho Chi Minh City, Vietnam

[3]Frantsevych Institute for Problems of Materials Science, National Academy of Sciences of Ukraine, 3 Krzhyzhanovsky Street, 03142 Kyiv, Ukraine

[4]Department of Experimental Physics and Information-Measuring Technology, Lesya Ukrainka Volyn National University, 13 Voli Avenue, 43025 Lutsk, Ukraine

[5]Department of Chemistry and Technology, Lesya Ukrainka Volyn National University, 13 Voli Avenue, 43025 Lutsk, Ukraine

[6]Jan Dlugosz University in Częstochowa, Armii Krajowej 13/15, PL-42-217 Częstochowa, Poland

[7]Department of Inorganic and Organic Chemistry, Lviv National University of Veterinary Medicine and Biotechnologies, 50 Pekarska Street, 79010 Lviv, Ukraine

[8]Inorganic Chemistry Department, Uzhhorod National University, 46 Pidhirna, UA-88000, Uzhhorod, Ukraine

Corresponding authors: khyzhun@ipms.kiev.ua, m.piasecki@ujd.edu.pl

Table S1. Crystallographic data for $Tl_2HgGeSe_4$

Space group : I -4 2 m

a = 7.9984(2) (Å)
c = 6.7645(2) (Å)
Cell volume = 432.76(4) ($Å^3$)
F(000) (electrons) = 820.0
Number of atoms in cell = 16.0
Calculated density = 7.6563(7) g/cm^3
Absorption coefficient (1/cm) = 1298.67
Radiation and wavelenght = CuKα 1.54185
Diffractometer = Powder
Mode of refinement = Full profile
Number of atom sites = 4
Number of free parameters = 16
Two-theta and sinT/l (max) : 100.02 0.497
h(min), k(min), l(min) : 0 0 0
h(max), k(max), l(max) : 6 8 7
R(intensity) : 0.0630 0.0823
Scale factor : 0.43991(6)
Texture axis and parameter : [2 2 0] 0.492(5)
Number of free parameters : 8

Atomic parameters for $Tl_2HgGeSe_4$

Atom	Wyck.	*x/a*	*y/b*	*z/c*	B(is/eq)
Hg	2*b*	0	0	1/2	2.70(3)
Tl	4*c*	0	1/2	0	1.87(2)
Ge	2*a*	0	0	0	1.04(6)
Se	8*i*	0.16558(8)	*x*	0.2164(2)	0.45(2)

Check CIF-file

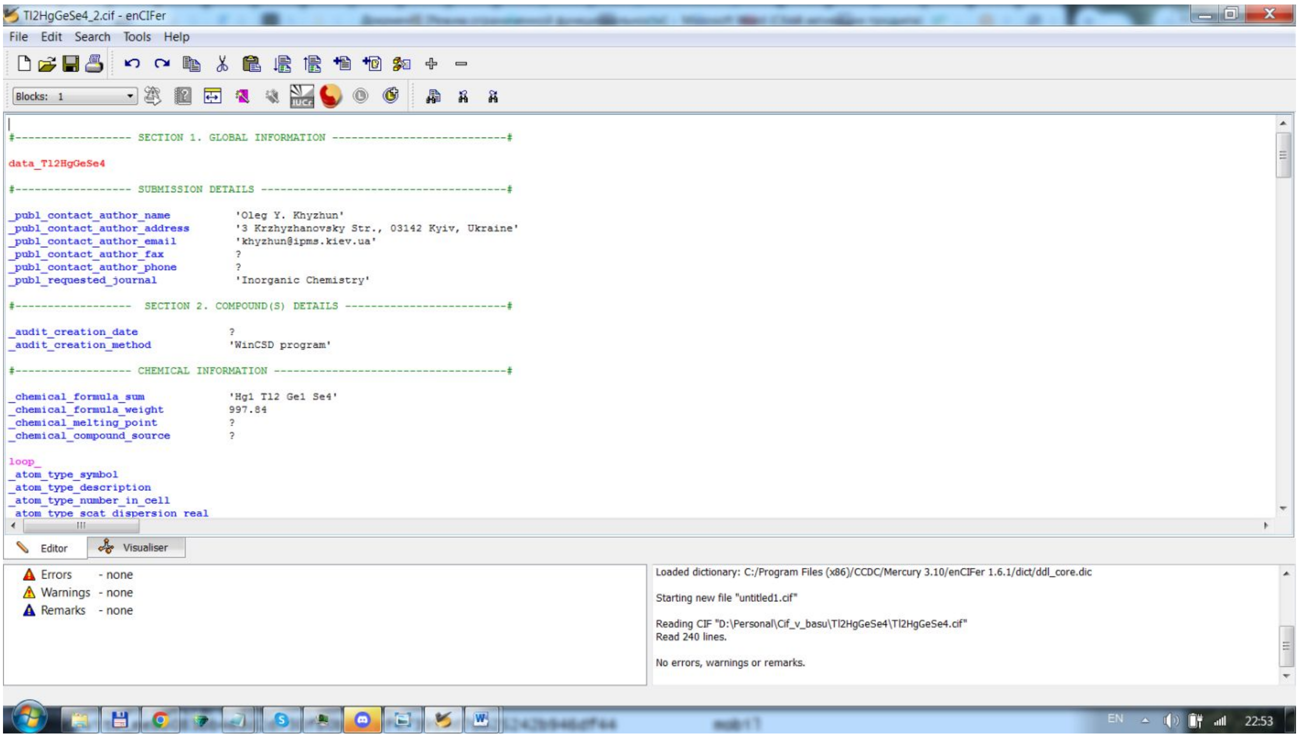

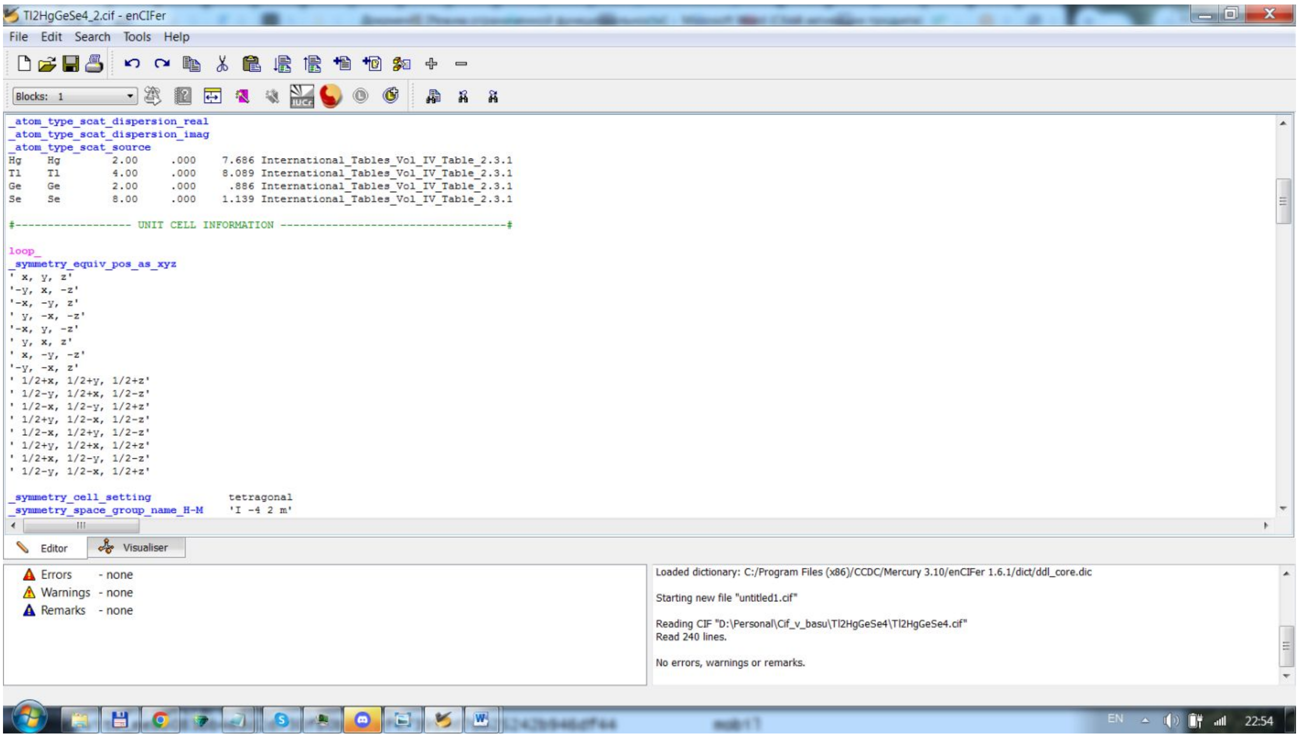

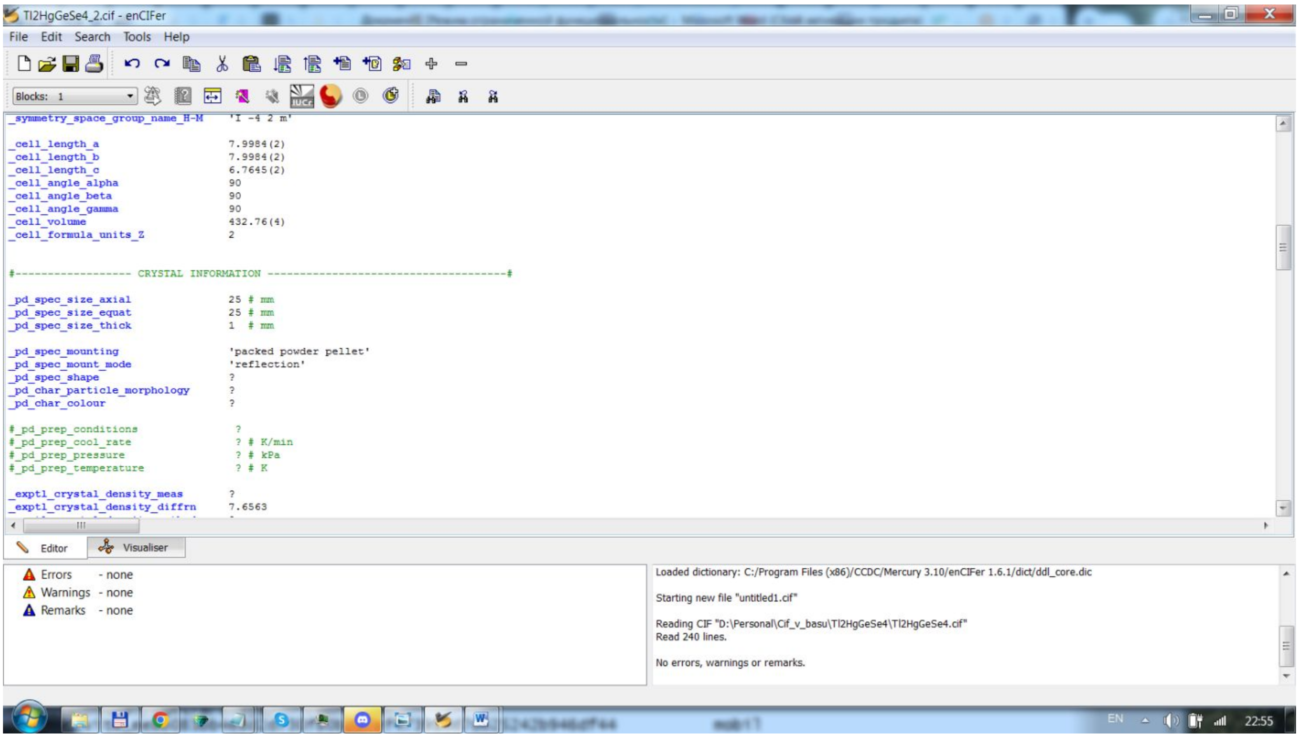

Tl2HgGeSe4_2.cif - enCIFer
File Edit Search Tools Help
Blocks: 1
_symmetry_space_group_name_H-M 'I -4 2 m'
_cell_length_a 7.9984(2)
_cell_length_b 7.9984(2)
_cell_length_c 6.7645(2)
_cell_angle_alpha 90
_cell_angle_beta 90
_cell_angle_gamma 90
_cell_volume 432.76(4)
_cell_formula_units_Z 2
#------------------ CRYSTAL INFORMATION ------------------------------------#
_pd_spec_size_axial 25 # mm
_pd_spec_size_equat 25 # mm
_pd_spec_size_thick 1 # mm
_pd_spec_mounting 'packed powder pellet'
_pd_spec_mount_mode 'reflection'
_pd_spec_shape ?
_pd_char_particle_morphology ?
_pd_char_colour ?
#_pd_prep_conditions ?
#_pd_prep_cool_rate ? # K/min
#_pd_prep_pressure ? # kPa
#_pd_prep_temperature ? # K
_exptl_crystal_density_meas ?
_exptl_crystal_density_diffrn 7.6563
Editor
Visualiser
Errors - none
Warnings - none
Remarks - none
Loaded dictionary: C:/Program Files (x86)/CCDC/Mercury 3.10/enCIFer 1.6.1/dict/ddl_core.dic
Starting new file "untitled1.cif"
Reading CIF "D:\Personal\Cif_v_basu\Tl2HgGeSe4\Tl2HgGeSe4.cif"
Read 240 lines.
No errors, warnings or remarks.
EN
22:55

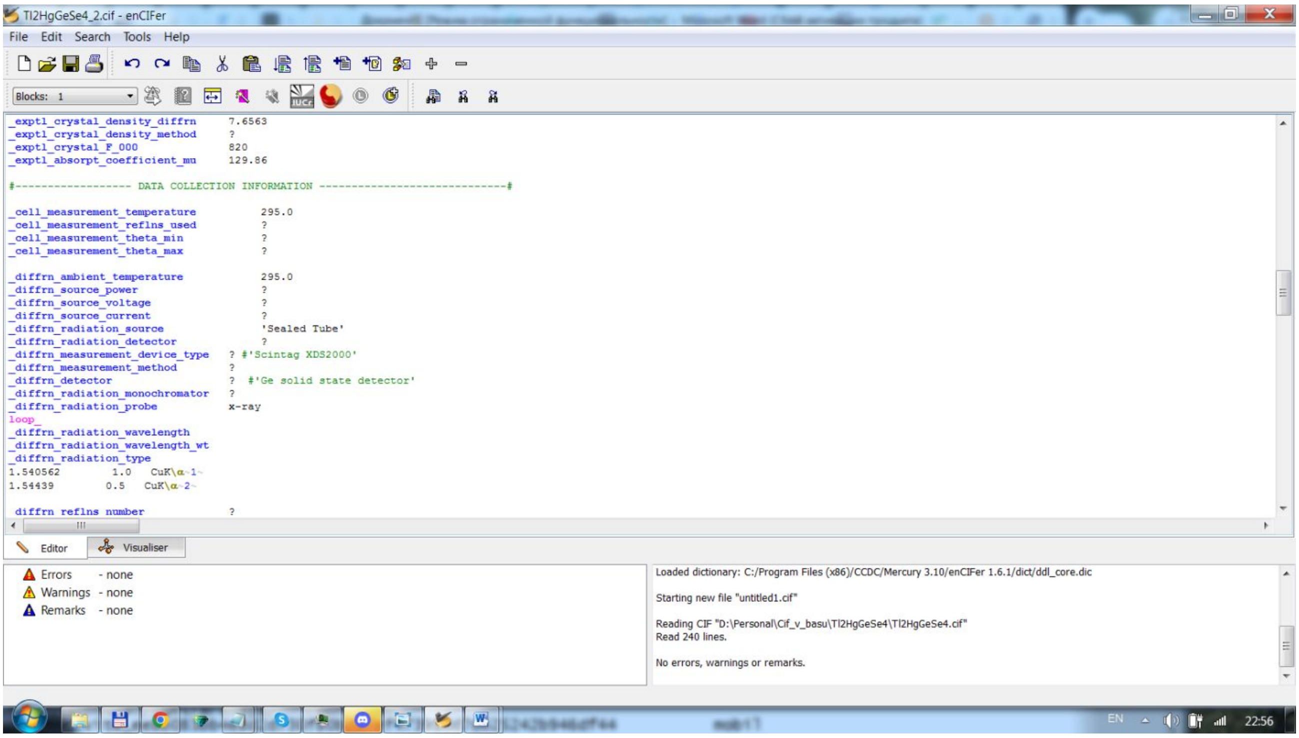

Tl2HgGeSe4_2.cif - enCIFer
File Edit Search Tools Help
Blocks: 1
_exptl_crystal_density_diffrn 7.6563
_exptl_crystal_density_method ?
_exptl_crystal_F_000 820
_exptl_absorpt_coefficient_mu 129.86
#------------------ DATA COLLECTION INFORMATION ----------------------------#
_cell_measurement_temperature 295.0
_cell_measurement_reflns_used ?
_cell_measurement_theta_min ?
_cell_measurement_theta_max ?
_diffrn_ambient_temperature 295.0
_diffrn_source_power ?
_diffrn_source_voltage ?
_diffrn_source_current ?
_diffrn_radiation_source 'Sealed Tube'
_diffrn_radiation_detector ?
_diffrn_measurement_device_type ? #'Scintag XDS2000'
_diffrn_measurement_method ?
_diffrn_detector ? #'Ge solid state detector'
_diffrn_radiation_monochromator ?
_diffrn_radiation_probe x-ray
loop_
_diffrn_radiation_wavelength
_diffrn_radiation_wavelength_wt
_diffrn_radiation_type
1.540562 1.0 CuK\a~1~
1.54439 0.5 CuK\a~2~
_diffrn_reflns_number ?
Editor
Visualiser
Errors - none
Warnings - none
Remarks - none
Loaded dictionary: C:/Program Files (x86)/CCDC/Mercury 3.10/enCIFer 1.6.1/dict/ddl_core.dic
Starting new file "untitled1.cif"
Reading CIF "D:\Personal\Cif_v_basu\Tl2HgGeSe4\Tl2HgGeSe4.cif"
Read 240 lines.
No errors, warnings or remarks.
EN
22:56

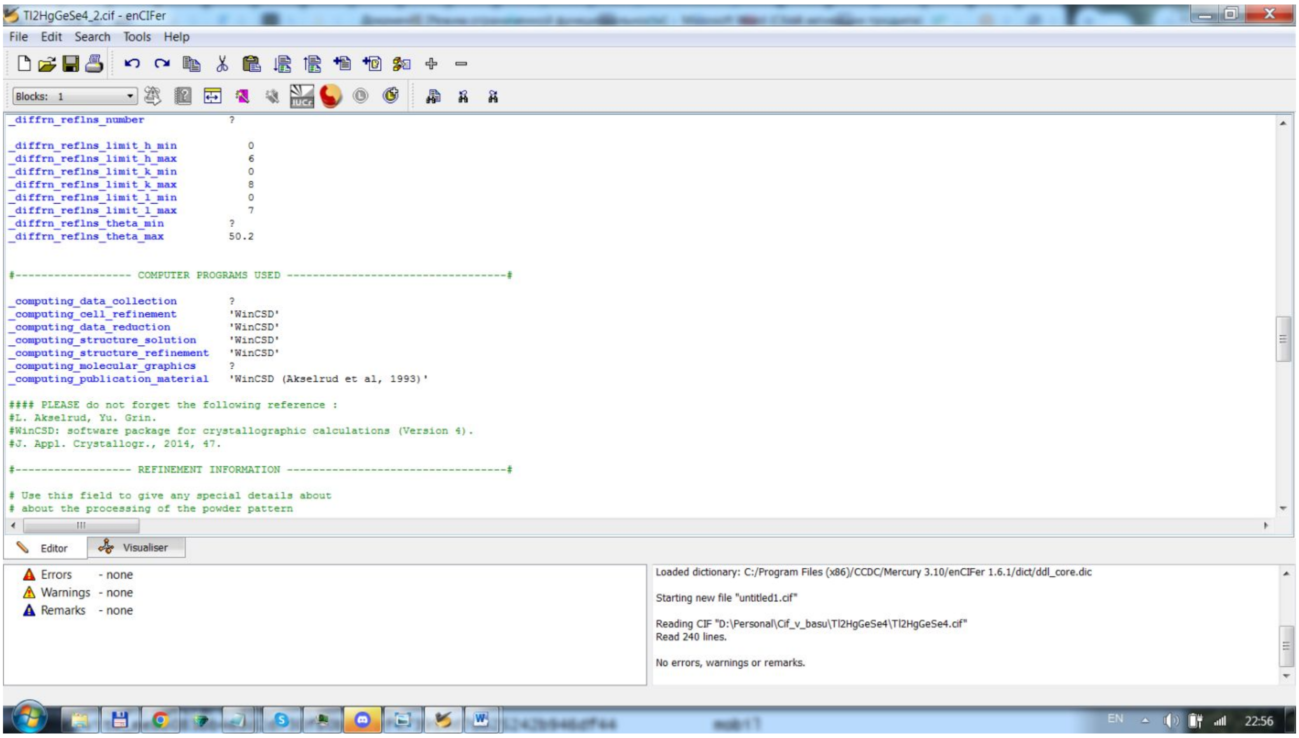
Tl2HgGeSe4_2.cif - enCIFer
File Edit Search Tools Help
Blocks: 1
_diffrn_reflns_number ?
_diffrn_reflns_limit_h_min 0
_diffrn_reflns_limit_h_max 6
_diffrn_reflns_limit_k_min 0
_diffrn_reflns_limit_k_max 8
_diffrn_reflns_limit_l_min 0
_diffrn_reflns_limit_l_max 7
_diffrn_reflns_theta_min ?
_diffrn_reflns_theta_max 50.2
#------------------ COMPUTER PROGRAMS USED -----------------------------------#
_computing_data_collection ?
_computing_cell_refinement 'WinCSD'
_computing_data_reduction 'WinCSD'
_computing_structure_solution 'WinCSD'
_computing_structure_refinement 'WinCSD'
_computing_molecular_graphics ?
_computing_publication_material 'WinCSD (Akselrud et al, 1993)'
PLEASE do not forget the following reference :
#L. Akselrud, Yu. Grin.
#WinCSD: software package for crystallographic calculations (Version 4).
#J. Appl. Crystallogr., 2014, 47.
#------------------ REFINEMENT INFORMATION -----------------------------------#
Use this field to give any special details about
about the processing of the powder pattern
Editor
Visualiser
Errors - none
Warnings - none
Remarks - none
Loaded dictionary: C:/Program Files (x86)/CCDC/Mercury 3.10/enCIFer 1.6.1/dict/ddl_core.dic
Starting new file "untitled1.cif"
Reading CIF "D:\Personal\Cif_v_basu\Tl2HgGeSe4\Tl2HgGeSe4.cif"
Read 240 lines.
No errors, warnings or remarks.
EN
22:56

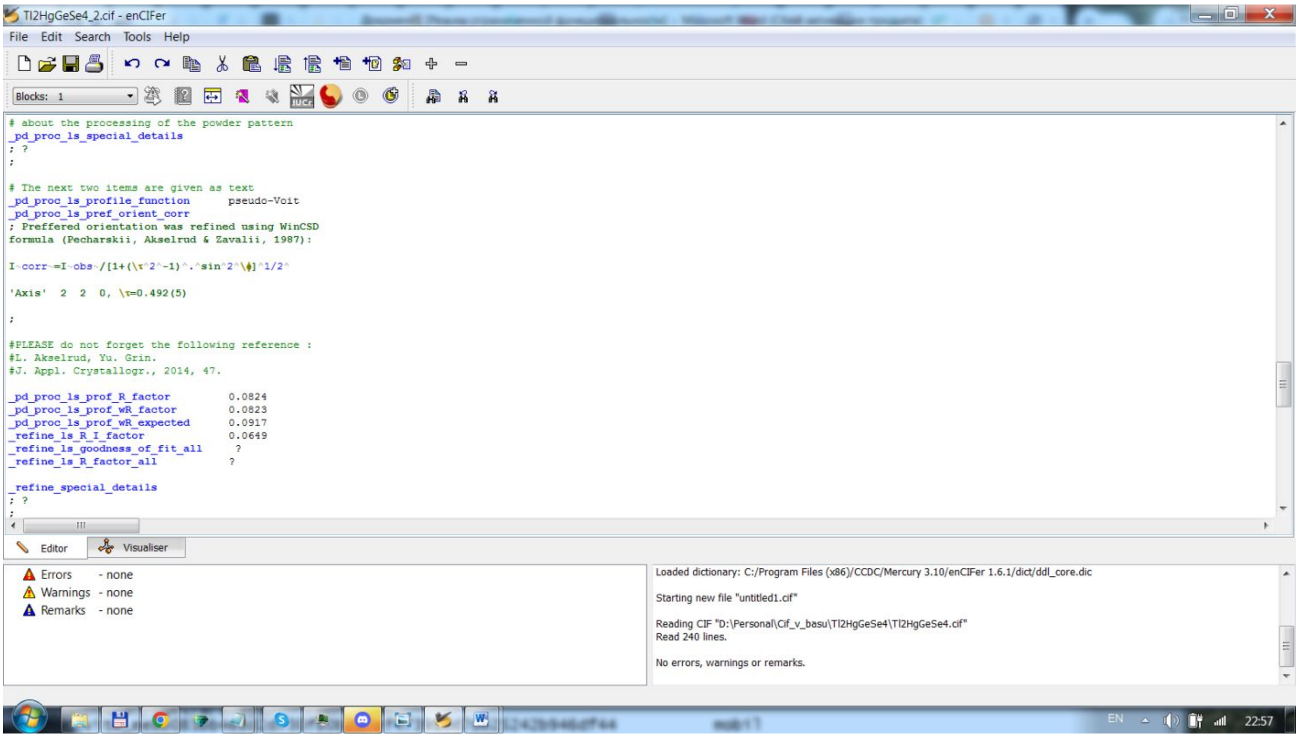
Tl2HgGeSe4_2.cif - enCIFer
File Edit Search Tools Help
Blocks: 1
about the processing of the powder pattern
_pd_proc_ls_special_details
; ?
;
The next two items are given as text
_pd_proc_ls_profile_function pseudo-Voit
_pd_proc_ls_pref_orient_corr
; Preffered orientation was refined using WinCSD
formula (Pecharskii, Akselrud & Zavalii, 1987):
I~corr~=I~obs~/[1+(\t^2^-1)^.^sin^2^\f]^1/2^
'Axis' 2 2 0, \t=0.492(5)
;
#PLEASE do not forget the following reference :
#L. Akselrud, Yu. Grin.
#J. Appl. Crystallogr., 2014, 47.
_pd_proc_ls_prof_R_factor 0.0824
_pd_proc_ls_prof_wR_factor 0.0823
_pd_proc_ls_prof_wR_expected 0.0917
_refine_ls_R_I_factor 0.0649
_refine_ls_goodness_of_fit_all ?
_refine_ls_R_factor_all ?
_refine_special_details
; ?
;
Editor
Visualiser
Errors - none
Warnings - none
Remarks - none
Loaded dictionary: C:/Program Files (x86)/CCDC/Mercury 3.10/enCIFer 1.6.1/dict/ddl_core.dic
Starting new file "untitled1.cif"
Reading CIF "D:\Personal\Cif_v_basu\Tl2HgGeSe4\Tl2HgGeSe4.cif"
Read 240 lines.
No errors, warnings or remarks.
EN
22:57

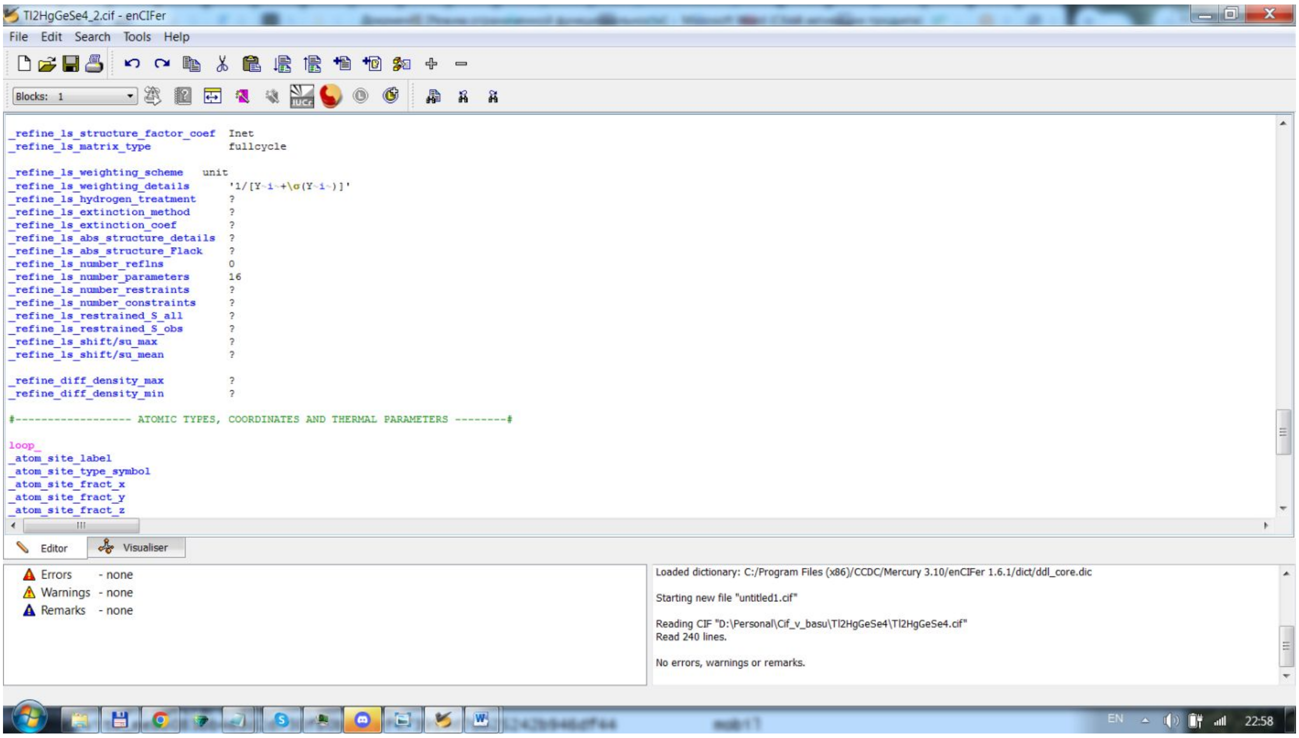
Tl2HgGeSe4_2.cif - enCIFer
File Edit Search Tools Help
Blocks: 1
_refine_ls_structure_factor_coef Inet
_refine_ls_matrix_type fullcycle
_refine_ls_weighting_scheme unit
_refine_ls_weighting_details '1/[Y~i~+\σ(Y~i~)]'
_refine_ls_hydrogen_treatment ?
_refine_ls_extinction_method ?
_refine_ls_extinction_coef ?
_refine_ls_abs_structure_details ?
_refine_ls_abs_structure_Flack ?
_refine_ls_number_reflns 0
_refine_ls_number_parameters 16
_refine_ls_number_restraints ?
_refine_ls_number_constraints ?
_refine_ls_restrained_S_all ?
_refine_ls_restrained_S_obs ?
_refine_ls_shift/su_max ?
_refine_ls_shift/su_mean ?
_refine_diff_density_max ?
_refine_diff_density_min ?
#----------------- ATOMIC TYPES, COORDINATES AND THERMAL PARAMETERS --------#
loop_
_atom_site_label
_atom_site_type_symbol
_atom_site_fract_x
_atom_site_fract_y
_atom_site_fract_z
Editor
Visualiser
Errors - none
Warnings - none
Remarks - none
Loaded dictionary: C:/Program Files (x86)/CCDC/Mercury 3.10/enCIFer 1.6.1/dict/ddl_core.dic
Starting new file "untitled1.cif"
Reading CIF "D:\Personal\Cif_v_basu\Tl2HgGeSe4\Tl2HgGeSe4.cif"
Read 240 lines.
No errors, warnings or remarks.
EN
22:58

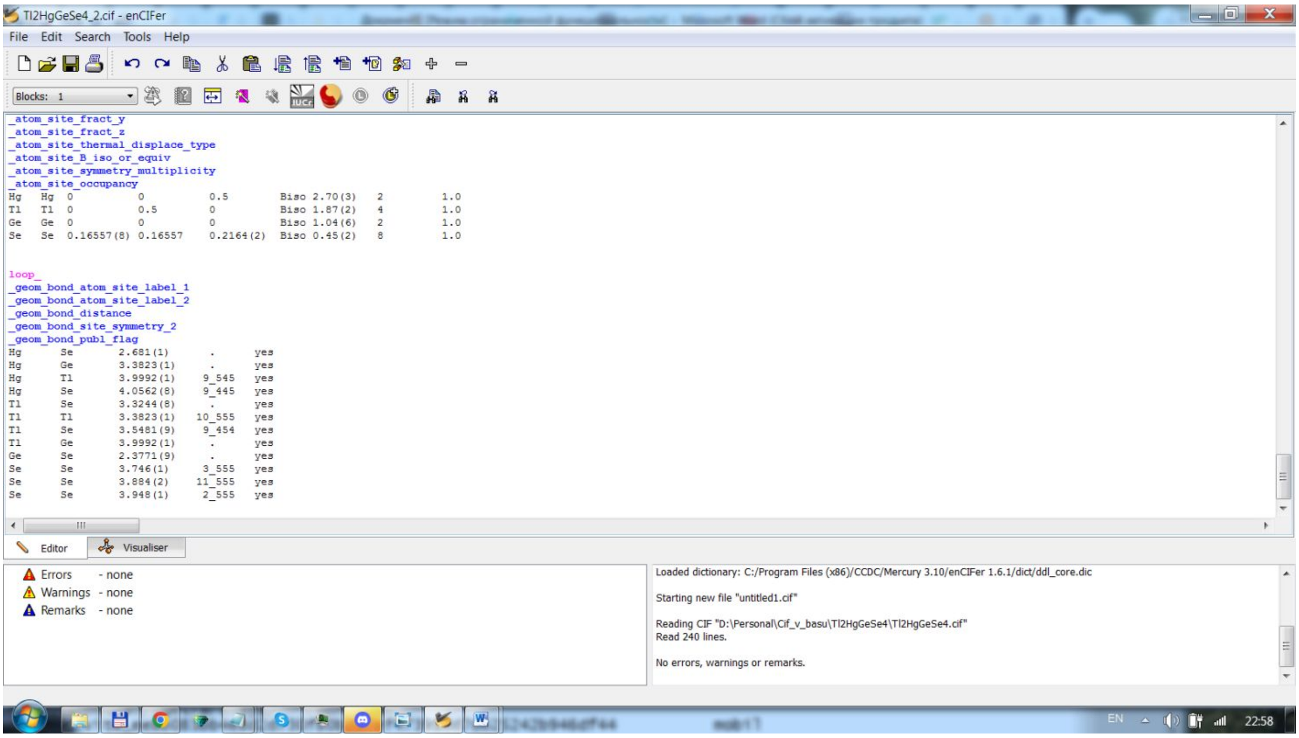
Tl2HgGeSe4_2.cif - enCIFer
File Edit Search Tools Help
Blocks: 1
_atom_site_fract_y
_atom_site_fract_z
_atom_site_thermal_displace_type
_atom_site_B_iso_or_equiv
_atom_site_symmetry_multiplicity
_atom_site_occupancy
Hg Hg 0 0 0.5 Biso 2.70(3) 2 1.0
Tl Tl 0 0.5 0 Biso 1.87(2) 4 1.0
Ge Ge 0 0 0 Biso 1.04(6) 2 1.0
Se Se 0.16557(8) 0.16557 0.2164(2) Biso 0.45(2) 8 1.0
loop_
_geom_bond_atom_site_label_1
_geom_bond_atom_site_label_2
_geom_bond_distance
_geom_bond_site_symmetry_2
_geom_bond_publ_flag
Hg Se 2.681(1) . yes
Hg Ge 3.3823(1) . yes
Hg Tl 3.9992(1) 9_545 yes
Hg Se 4.0562(8) 9_445 yes
Tl Se 3.3244(8) . yes
Tl Tl 3.3823(1) 10_555 yes
Tl Se 3.5481(9) 9_454 yes
Tl Ge 3.9992(1) . yes
Ge Se 2.3771(9) . yes
Se Se 3.746(1) 3_555 yes
Se Se 3.884(2) 11_555 yes
Se Se 3.948(1) 2_555 yes
Editor
Visualiser
Errors - none
Warnings - none
Remarks - none
Loaded dictionary: C:/Program Files (x86)/CCDC/Mercury 3.10/enCIFer 1.6.1/dict/ddl_core.dic
Starting new file "untitled1.cif"
Reading CIF "D:\Personal\Cif_v_basu\Tl2HgGeSe4\Tl2HgGeSe4.cif"
Read 240 lines.
No errors, warnings or remarks.
EN
22:58
